# Vascular Calcifying Progenitor Cells Possess Bidirectional Differentiation Potentials

**DOI:** 10.1371/journal.pbio.1001534

**Published:** 2013-04-09

**Authors:** Hyun-Ju Cho, Hyun-Jai Cho, Ho-Jae Lee, Myung-Kang Song, Ji-Yun Seo, Yeon-Hee Bae, Ju-Young Kim, Hae-Young Lee, Whal Lee, Bon-Kwon Koo, Byung-Hee Oh, Young-Bae Park, Hyo-Soo Kim

**Affiliations:** 1 National Research Laboratory for Stem Cell Niche, Seoul National University College of Medicine, Seoul, Korea; 2 Cardiovascular Center & Department of Internal Medicine, Seoul National University Hospital, Seoul, Korea; 3 Innovative Research Institute for Cell Therapy, Seoul National University Hospital, Seoul, Korea; 4 Department of Radiology, Seoul National University College of Medicine, Seoul, Korea; 5 World Class University Program, Department of Molecular Medicine and Biopharmaceutical Sciences, Seoul National University, Seoul, Korea; Howard Hughes Medical Institute, The Salk Institute for Biological Studies, United States of America

## Abstract

Vascular calcification is an advanced feature of atherosclerosis for which no effective therapy is available. To investigate the modulation or reversal of calcification, we identified calcifying progenitor cells and investigated their calcifying/decalcifying potentials. Cells from the aortas of mice were sorted into four groups using Sca-1 and PDGFRα markers. Sca-1^+^ (Sca-1^+^/PDGFRα^+^ and Sca-1^+^/PDGFRα^−^) progenitor cells exhibited greater osteoblastic differentiation potentials than Sca-1^−^ (Sca-1^−^/PDGFRα^+^ and Sca-1^−^/PDGFRα^−^) progenitor cells. Among Sca-1^+^ progenitor populations, Sca-1^+^/PDGFRα^−^ cells possessed bidirectional differentiation potentials towards both osteoblastic and osteoclastic lineages, whereas Sca-1^+^/PDGFRα^+^ cells differentiated into an osteoblastic lineage unidirectionally. When treated with a peroxisome proliferator activated receptor γ (PPARγ) agonist, Sca-1^+^/PDGFRα^−^ cells preferentially differentiated into osteoclast-like cells. Sca-1^+^ progenitor cells in the artery originated from the bone marrow (BM) and could be clonally expanded. Vessel-resident BM-derived Sca-1^+^ calcifying progenitor cells displayed nonhematopoietic, mesenchymal characteristics. To evaluate the modulation of in vivo calcification, we established models of ectopic and atherosclerotic calcification. Computed tomography indicated that Sca-1^+^ progenitor cells increased the volume and calcium scores of ectopic calcification. However, Sca-1^+^/PDGFRα^−^ cells treated with a PPARγ agonist decreased bone formation 2-fold compared with untreated cells. Systemic infusion of Sca-1^+^/PDGFRα^−^ cells into Apoe^−/−^ mice increased the severity of calcified atherosclerotic plaques. However, Sca-1^+^/PDGFRα^−^ cells in which PPARγ was activated displayed markedly decreased plaque severity. Immunofluorescent staining indicated that Sca-1^+^/PDGFRα^−^ cells mainly expressed osteocalcin; however, activation of PPARγ triggered receptor activator for nuclear factor-κB (RANK) expression, indicating their bidirectional fate in vivo. These findings suggest that a subtype of BM-derived and vessel-resident progenitor cells offer a therapeutic target for the prevention of vascular calcification and that PPARγ activation may be an option to reverse calcification.

## Abstract 2

Calcifying progenitor cells in blood vessels have the potential to differentiate into cells that either promote calcium accumulation or reverse accumulation, and treatment with PPAR? can shift the direction of this differentiation.

## Author Summary

Atherosclerosis involves hardening of the arteries and can lead to heart disease. Calcium accumulation in blood vessels contributes to this process, and this process is regulated by cells that promote calcium accumulation (osteoblasts) and cells that reverse the accumulation (osteoclasts). In this study, we show that vascular calcifying progenitor cells in the blood vessel have the potential to become either osteoblasts or osteoclasts, and that a drug can push these cells towards becoming osteoclasts instead of osteoblasts. Progenitor cells that express both Sca-1 and PDGFRα cell surface proteins were more committed to differentiate into osteoblasts, while cells that only expressed Sca-1 could differentiate into osteoblasts or osteoclasts in a bidirectional manner. Moreover, treatment with a PPARγ agonist could shift the direction of differentiation of Sca-1^+^/PDGFRα^−^ progenitor cells toward osteoclast-like cells, whereas it cannot influence the fates of Sca-1^+^/PDGFRα^+^ progenitors. These results offer new therapeutic targets for reversing calcium accumulation in blood vessels.

## Introduction

Vascular calcification (VC) is a feature of progressive and advanced atherosclerosis that is regarded as a prognostic marker of adverse cardiovascular events [1],[2]. No therapies are available to ameliorate VC [3]. The pathophysiology of VC involves a strict and active regulatory process that resembles bone formation [4] and functions to maintain a balance between osteoblastic and osteoclastic cells [5]. The origin of osteoblastic cells in the vasculature remains an issue of active debate [6]. Resident vascular smooth muscle cells (VSMCs) and calcifying vascular cells have been examined to elucidate the cellular origins of VC. Pericytes, mesenchymal stem cells (MSCs), myofibroblasts, and circulating osteoprogenitor cells have been isolated from the vasculature and shown to have osteoblastic potential [7]–[10]. However, few studies have addressed the origins, features, and roles of osteoclastic and decalcifying cells in the vasculature or the balance between osteoblastic and osteoclastic cells during VC.

In this study, we aimed to identify vascular calcifying progenitor cells and to modulate or reverse VC. We first isolated vessel-resident calcifying progenitor cells using stem cell antigen-1 (Sca-1) and platelet-derived growth factor receptor alpha (PDGFRα) antibodies in the vasculature. We then identified a population of nonhematopoietic mesenchymal Sca-1^+^ cells (Sca-1^+^/PDGFRα^+^ and Sca-1^+^/PDGFRα^−^ cells) that originated from the bone marrow (BM) and could be clonally expanded. Among the Sca-1^+^ populations, Sca-1^+^/PDGFRα^+^ cells possessed unidirectional osteoblastic potential. In contrast, Sca-1^+^/PDGFRα^−^ cells possessed bidirectional osteoblastic and osteoclastic differentiation potentials. Both calcifying progenitor Sca-1^+^/PDGFRα^+^ cells and Sca-1^+^/PDGFRα^−^ cells induced ectopic mineralization and atherosclerotic calcification in vivo. When PPARγ was activated in bidirectional Sca-1^+^/PDGFRα^−^ cells, calcium accumulation was reduced, and plaque severity was decreased. This cell population may offer new therapeutic targets and modalities for ameliorating VC.

## Results

### Osteoblastic and Osteoclastic Differentiation Potentials of Progenitor Cells in the Vasculature

**Figure 1:**
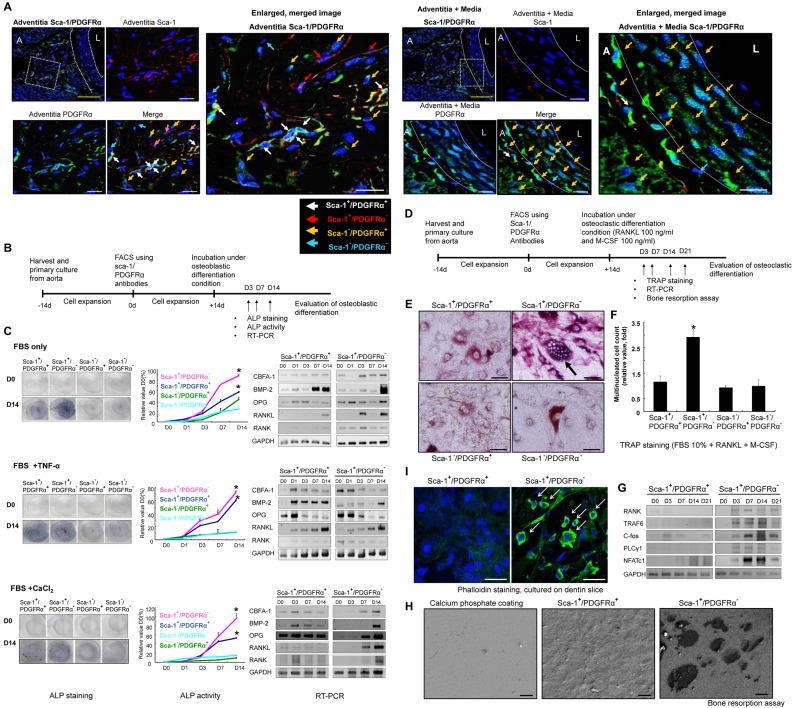
Osteoblastic and osteoclastic differentiation potentials of calcifying progenitor cells in the vasculature. (A) Sca-1 (red) and PDGFRα (green) immunostaining to detect vascular calcifying progenitor cells from WT C57 mouse aortas (*n* = 5). The three panels on the right depict high-magnification images of the white squares indicated on the left. The white dashed lines specify the culture media. L, lumen; A, adventitia; Blue, Sytox Blue nuclear staining. Bars: yellow = 100 µm; white = 20 µm. (B) Schematic of the experiments indicating the three different conditions of osteoblast induction. (C) Cells were cultured with 10% FBS; 10% FBS+10 ng/ml TNF-α; or 10% FBS+1.25 mM CaCl_2_+2 mM β-glycerolphosphate. Compared with Sca-1^−^ cells, Sca-1^+^ cells cultured under osteoblastic conditions showed significantly higher numbers of ALP positive cells, greater ALP activity, and higher expression levels of osteoblast-related genes. *P<0.01 versus Sca-1^−^/PDGFRα^+^ cells. Under moderate osteoblastic induction conditions (FBS only and FBS+CaCl_2_), Sca-1^+^/PDGFRα^−^ cells expressed both RANK and RANKL. Under the most potent osteoblastic differentiation conditions (FBS+TNF-α), RANK expression was not induced. Experiments were performed in triplicate. (D) Schematic of the experiments of osteoclast induction. (E and F) Under osteoclastic differentiation conditions, Sca-1^+^/PDGFRα^−^ cells differentiated into TRAP-positive, multinucleated cells (>3 nuclei; *n* = 5 per group). *P<0.001 versus Sca-1^−^/PDGFRα^+^ cells after 7 d of differentiation. Bars: 50 µm. (G) Osteoclast-related genes were upregulated in Sca-1^+^/PDGFRα^−^ cells. (H) Calcifying progenitor cells were cultured on calcium phosphate-coated discs. SEM imaging indicated that Sca-1^+^/PDGFRα^−^ cells generated a typical resorption area (calcium pore size) in contrast to Sca-1^+^/PDGFRα^+^ cells. Bars: 10 µm. (I) Under the same conditions in (H), Sca-1^+^/PDGFRα^−^ cells formed dual actin sealing zones as demonstrated by FITC-conjugated phalloidin staining. Bars: 50 µm.

To identify putative calcifying progenitor cells, we stained tissue sections with stem/progenitor markers [11]. We detected marker-positive cells, particularly Sca-1^+^ cells, in the artery (Figure S1). Sca-1 is a marker of hematopoietic stem cells [12] and MSCs [13] in mice. To distinguish among progenitor cells in the vasculature, we also stained for PDGFRα [14]. Both Sca-1^+^ and PDGFRα^+^ cells were detected in the artery (Figure 1A).

Our double-immunostaining enabled us to categorize cells into the following four groups: Sca-1^+^/PDGFRα^+^, Sca-1^+^/PDGFRα^−^, Sca-1^−^/PDGFRα^+^, and Sca-1^−^/PDGFR^−^. We subsequently isolated and propagated aortic cells (Figure S2) and performed fluorescence-activated cell sorting (FACS). No difference in sorting was detected between cells sorted immediately (Figure S3) or after 2 wk of cell expansion. We confirmed the purities of the sorted cell populations by immunostaining (Figure S2C).

We next assessed the osteoblastic differentiation potentials of the four cell groups over time (Figure 1B). Under three osteoblastic differentiation conditions [15], Sca-1^+^ cells (Sca-1^+^/PDGFRα^+^, Sca-1^+^/PDGFRα^−^) showed significantly higher numbers and activities of alkaline phosphatase (ALP)–positive cells than Sca-1^−^ cells (Sca-1^−^/PDGFRα^+^, Sca-1^−^/PDGFR^−^). We also confirmed the level of mRNA expression of several osteoblast-related genes in the four groups of cells (Figure 1C and Figure S4A,B). Our results indicate that Sca-1^+^ cells are superior to Sca-1^−^ cells in terms of osteoblastic differentiation potentials and osteoblastic fate adaptation capacities.

We also determined the osteoclastic potentials of the four cell groups. The cells were cultured in osteoclast differentiation media (Figure 1D). Osteoclastic differentiation potentials were then assessed by tartrate-resistant acidic phosphatase (TRAP) staining [16],[17]. The Sca-1^+^/PDGFRα^−^ cell population was comprised of significantly more TRAP-positive cells than the other three cell groups (Figure 1E,F). Under moderate osteoblastic differentiation conditions, Sca-1^+^/PDGFRα^−^ cells highly expressed the osteoclast-related gene, receptor activator for nuclear factor-κB (RANK), and the osteoblast-related genes, osteoprotegerin (OPG), and RANK ligand (RANKL). However, in serum containing tumor necrosis factor-α (TNF-α), RANK expression was suppressed (Figure 1C). We detected the expression of other osteoclast-related genes in Sca-1^+^/PDGFRα^−^ cells, but not in Sca-1^+^/PDGFRα^+^ cells (Figure 1G and ).

To examine whether differentiated Sca-1^+^/PDGFRα^−^ cells function as osteoclast-like cells, we measured calcium resorption ability. Cells were cultured on a calcium phosphate-coated disc treated with RANKL and macrophage-colony stimulating factor (M-CSF). Observation of the discs by scanning electronic microscopy (SEM) indicated that Sca-1^+^/PDGFRα^−^ cells generated wider areas of calcium resorption and greater pore sizes than Sca-1^+^/PDGFRα^+^ cells (Figure 1H). We assayed for the formation of dual actin ring sealing zones [18]. Under osteoclastic differentiation conditions, Sca-1^+^/PDGFRα^−^ cells cultured on dentine slices formed actin ring of the sealing zones characteristic of active osteoclasts (Figure 1I). These structures were absent in Sca-1^+^/PDGFRα^+^ cells cultured under the same conditions. These results confirm that Sca-1^+^/PDGFRα^−^ cells have the potential to differentiate into functioning osteoclast-like cells. Sca-1^+^/PDGFRα^−^ cells possess bidirectional differentiation potentials toward both the osteoblastic and osteoclastic lineages, whereas Sca-1^+^/PDGFRα^+^ cells have a unidirectional differentiation potential toward the osteoblastic lineage.

We tested the fates of Sca-1^+^/PDGFRα^+^ and Sca-1^+^/PDGFRα^−^ cells in mixed medium containing FBS and TNF-α (osteoblastic differentiation stimulators) with RANKL and M-CSF (osteoclastic differentiation stimulators). We examined differentiation potentials by immunofluorescent staining of osteocalcin and cathepsin K. Seven days after incubation in mixed medium, both Sca-1^+^/PDGFRα^+^ and Sca-1^+^/PDGFRα^−^ cells dominantly expressed osteocalcin. Interestingly, a few Sca-1^+^/PDGFRα^−^ cells expressed cathepsin K but did not form multinucleated cells. Sca-1^+^/PDGFRα^+^ cells did not express cathepsin K. These data suggest that the fates of bidirectional Sca-1^+^/PDGFRα^−^ cells could be primarily osteoblastic in the mixed in vivo environment (Figure S5).

Because Sca-1^+^/PDGFRα^+^ cells do not possess bidirectional differentiation capacities, we examined whether PDGFRα directly influences the differentiation phenotypes of these cells. We treated Sca-1^+^/PDGFRα^+^ cells with PDGF-BB (10 ng/ml or 25 ng/ml) and 10% charcoal-stripped FBS for 0, 3, 7, or 14 d. TNF-α treatment was used as a positive stimulation control in flow cytometry. We analyzed osteoblast-related gene expression by real-time PCR and ALP expression by flow cytometry. The mRNA expression levels of osteoblast-related genes were mildly increased in the PDGF-BB treatment group compared with the FBS only treatment group. Similarly, FACS indicated that PDGF-BB treatment marginally increased ALP expression compared with FBS only. suggesting that PDGFRα is not a functional receptor for osteoblastic differentiation. In contrast, TNF-α treatment markedly increased ALP expression. In PDGFRα^+^ cells, PDGF-BB mildly stimulated osteoblast differentiation and promoted cells to migrate into artificial bone structures [19],[20]. These researchers as well as we concluded that PDGFRα was a marker but not a functional receptor for osteoblastogenesis (Figure S6).

### Single Clonal Expansion of Calcifying Progenitor Cells

**Figure 2:**
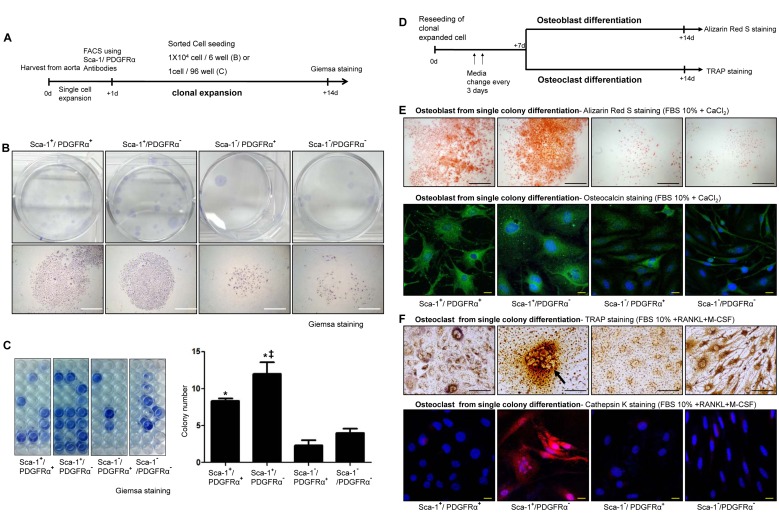
Clonal expansion of calcifying progenitor cells. (A) Schematic of the clonal expansion assay. (B) Giemsa staining to detect a single-cell colony. Bars: 1 mm. (C) For statistical analysis, colony-forming cells were counted among 96 wells per group. Experiments were performed in triplicate. Colonies formed by Sca-1^+^ cells were much more compact and abundant than Sca-1^−^ cells. *P<0.001 versus Sca-1^−^/PDGFRα^+^ cells. ‡*P*<0.005 versus Sca-1^+^/PDGFRα^+^ cells. (D) Schematic depicting osteoblastic and osteoclastic differentiation of clonally expanded cells. (E) Alizarin Red S and osteocalcin staining to detect osteoblast differentiation from single-colony cells after 14 d of differentiation. Bars: black = 1 mm; white = 20 µm. (F) TRAP and cathepsin K staining to detect osteoclast differentiation from single-colony cells after 14 d of differentiation. Bars: black = 100 µm; white = 20 µm.

To test the capacity of calcifying progenitor cells from single clones, we performed a clonal expansion assay (Figure 2A). Sca-1^+^ cells generated compact and abundant colonies and exhibited a greater colony-forming efficiency than Sca-1^−^ cells, which barely generated any colonies (Figure 2B,C). We also investigated whether calcifying progenitor cells derived from a single colony could differentiate into either osteoblastic or osteoclastic cells (Figure 2D). Single colony-derived Sca-1^+^ cells differentiated into osteoblasts (Figure 2E). Sca-1^+^/PDGFRα^−^ clonally expanded cells differentiated into multinucleated osteoclast-like cells under osteoclastic differentiation conditions (Figure 2F), suggesting that this cell population was comprised of bidirectional calcifying progenitor cells with both osteoblastic and osteoclastic differentiation potentials.

### The Origin of Calcifying Progenitor Cells in the Artery

**Figure 3:**
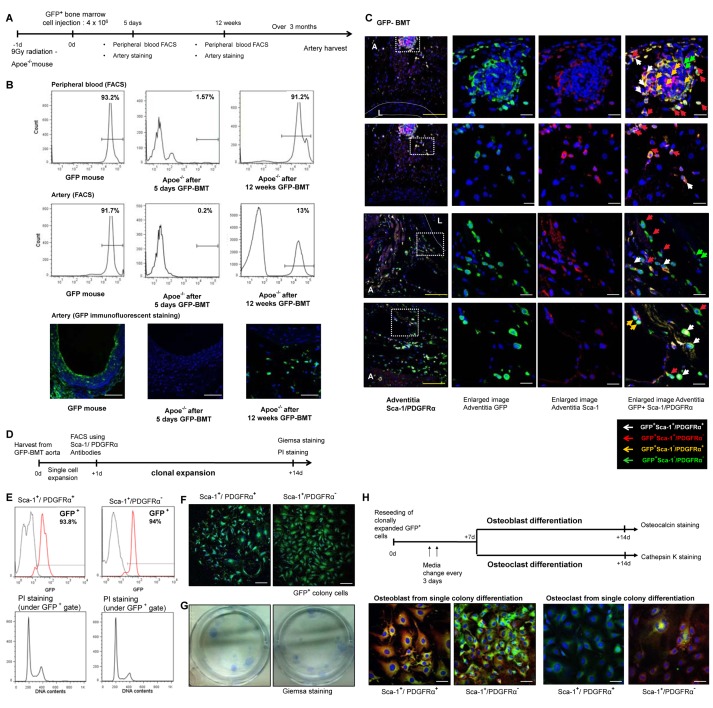
The origin of calcifying progenitor cells. (A) BMT experimental outline. (B) After 5 d of cell infusion, we checked the presence of GFP+ cells in arteries and blood using FACS and immunofluorescent staining. A small fraction of donor GFP+ cells were detected in peripheral blood (1.5%) but were rarely detected in the artery (0.2%). Twelve weeks after transplantation GFP+ cells from donor BM were reconstituted with peripheral blood cells of C57 mice (up to 90%). At that time, 13% of arterial resident cells were GFP+. Thus, the majority of GFP+ cells gradually were incorporated into the artery within a considerable duration. Bars: 50 µm. (C) Aortas were harvested after 6 mo of BMT and were stained with antibodies targeting GFP, Sca-1, or PDGFRα. The three panels on the right depict high-magnification images of the white squares shown on the left. Blue, Sytox Blue nuclear staining; L, lumen; A, adventitia. White dashed lines describe the media. Bars: yellow = 100 µm; white = 20 µm. (D) Schematic of the GFP^+^ clonal expansion assay. (E) PI staining to identify fusion between GFP^+^ and non-GFP^+^ cells. (F) GFP^+^ single clone immunofluroscent staining. Bars: 200 µm. (G) Giemsa staining of single-cell colonies. (H) Osteocalcin/cathepsin K staining of osteoblast/osteoclast differentiation from GFP^+^ single colonies after 14 d.

Osteoblasts and osteoblastic progenitor cells have been well-characterized in the bone and BM [21],[22]. To determine whether calcifying progenitor cells originate in BM, we performed a BM transplantation (BMT) experiment using GFP mice as a marker (Figure 3A).

Five days after cell infusion, to rule out the possibility that cells migrated from the intravascular space to vessels, we examined the presence of GFP^+^ cells in the arteries and blood using FACS and immunofluorescent staining. A few GFP^+^ cells from the donor were detected in the peripheral blood (1.5%), but these were rarely detected in the artery (0.2%), indicationg that GFP^+^ cells from donor marrow did not exhibit diapedesis directly into the arterial wall. Twelve weeks after transplantiation (Figure 3B), GFP^+^ cells from donor BM reconstituted blood cells in C57 background mice comprised up to 90% of peripheral blood cells. At that point, 13% of arterial resident cells were GFP^+^. Takgen together, these data indicate that the majority of GFP^+^ cells were gradually incorporated into the artery in a considerable amount of time.

We then determined vessel infiltration of BM-derived GFP^+^Sca-1^+^ cells by immunostaining in the artery (Figure 3C). We also assessed the possibility of the fusion between BM-derived GFP^+^ cells and non-BM cells using propidium iodide (PI) staining. GFP^+^ cells with DNA contents beyond 4n were not detected (Figure 3D,E).

We performed a GFP^+^ clonal expansion assay in BM-derived GFP^+^Sca-1^+^/PDGFRα^+^ and GFP^+^Sca-1^+^/PDGFRα^−^ cell populations from the vessel walls of chimeric mice. GFP^+^Sca-1^+^ cells (GFP^+^Sca-1^+^/PDGFRα^+^ or GFP^+^Sca-1^+^/PDGFRα^−^ cells) were capable of colony generation (Figure 3F,G). Single colony derived GFP^+^Sca-1^+^/PDGFRα^−^ cells expanded from a single colony possessed both osteoblastic and osteoclastic differentiation potentials (Figure 3H).

### BM-Derived Vessel Resident Calcifying Progenitor Cells Are Nonhematopoietic and Mesenchymal

We subsequently characterized BM-derived and vessel-resident calcifying progenitor cells. Aortas were harvested from Apoe^−/−^ mice that underwent GFP–BMT. BM-derived GFP^+^ cells were negative for a hematopoietic lineage antibody cocktail (Lin^−^) containing antibodies targeting CD3, CD11b (monophage/macrophage marker), CD45R/B220, TER-11, and Ly-6G.

**Figure 4:**
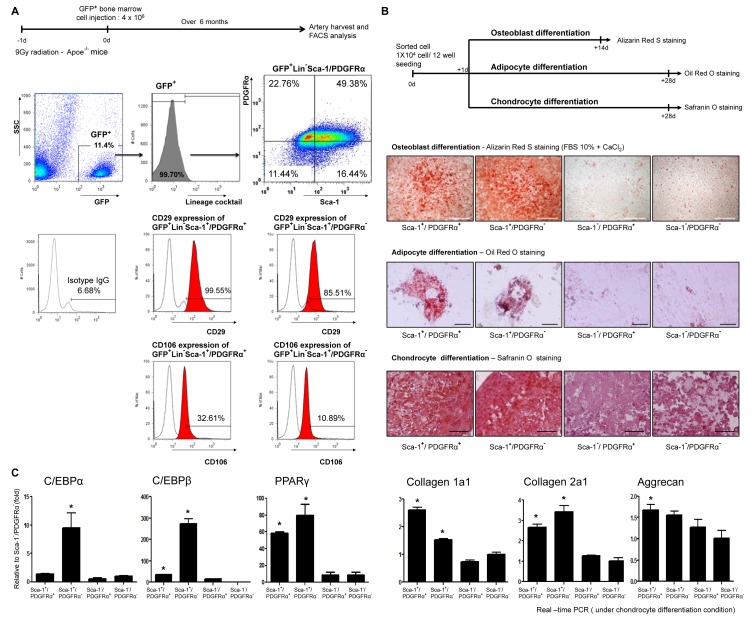
Vessel resident calcifying progenitor cells are mesenchymal but not hematopoietic. (A) FACS of arterial cells from GFP–BMT Apoe^−/−^ mice. GFP^+^ cells were negative for Lineage antibody cocktail targets. GFP^+^Lin^−^Sca-1^+^/PDGFRα^+^ or GFP^+^Lin^−^Sca-1^+^/PDGFRα^−^ cells expressed CD29 or CD106 (*n* = 10, performed in triplicate). (B) Schematic of osteoblast, adipocyte, and chondrocyte inductions from calcifying progenitor cells. Cells were stained with Alizarin Red S after 14 d of differentiation; bars: 1 mm. Oil Red O staining 28 d after differentiation; bars: 50 µm. Safranin O staining 28 d after differentiation; bars: 50 µm. (C) Adipocyte-related genes and chondrocyte-related genes were upregulated in Sca-1^+^ cells (Sca-1^+^/PDGFRα^+^, Sca-1^+^/PDGFRα^−^) under each differentiation condition.

Calcifying progenitor cells then were isolated from GFP^+^Lin^−^ cells by detecting Sca-1 and PDGFRα expression. GFP^+^Lin^−^Sca-1^+^/PDGFRα^+^ cells highly expressed the MSC markers, CD29 and CD106, and GFP^+^Lin^−^Sca-1^+^/PDGFRα^−^ cells weakly expressed CD29 and CD106. These results suggest that BM-derived calcifying progenitor cells have characteristics of MSCs but not of hematopoietic cells or of contaminating monocytes/macrophages (Figure 4A).

We next isolated vessel-resident calcifying progenitor cells and assessed their differentiation potentials as MSCs. Sca-1^+^ cells were capable of differentiating into osteoblasts, adipocytes, and chondrocytes (Figure 4B). We measured the expression levels of the following adipocyte- and chondrocyte-specific genes by real-time PCR. Under each differentiation condition, Sca-1^+^ cells (Sca-1^+^/PDGFRα^+^ and Sca-1^+^/PDGFRα^−^) showed significantly higher adipocyte- and chondrocyte-specific gene expression levels, as compared with Sca-1^−^ cells (Sca-1^−^/PDGFRα^+^ and Sca-1^−^/PDGFR^−^). These results indicate that Sca-1^+^ cells have a nonhematopoietic, MSC-like nature (Figure 4C).

### Ex Vivo Osteoblastic and Osteoclastic Differentiation of BM-Derived Vascular Calcifying Progenitor Cells and the Modulation of Differentiation by a PPARγ Agonist

To confirm the ex vivo osteoblastic and osteoclastic differentiation abilities of BM-derived calcifying progenitor cells, we harvested and cultured cells from the aortas of C57 mice that had undergone GFP–BMT (Figure S7A). Cultured aortic cells were divided into four groups of GFP^+^ cells with respect to their Sca-1/PDGFRα statuses and were used in osteoblastic and osteoclastic differentiation experiments.

Under osteoblastic differentiation conditions, GFP^+^Sca-1^+^ cells differentiated more readily into osteoblasts than GFP^+^Sca-1^−^ cells. Under osteoclastic differentiation conditions, GFP^+^Sca-1^+^/PDGFRα^−^ cells exclusively differentiated into osteoclasts. These findings suggest that BM-derived vessel-resident Sca-1^+^/PDGFRα^−^ cells possess osteoblastic/osteoclastic differentiation potentials. In contrast, GFP^+^Sca-1^+^/PDGFRα^+^ cells displayed only osteoblastic differentiation (Figure S7).

**Figure 5:**
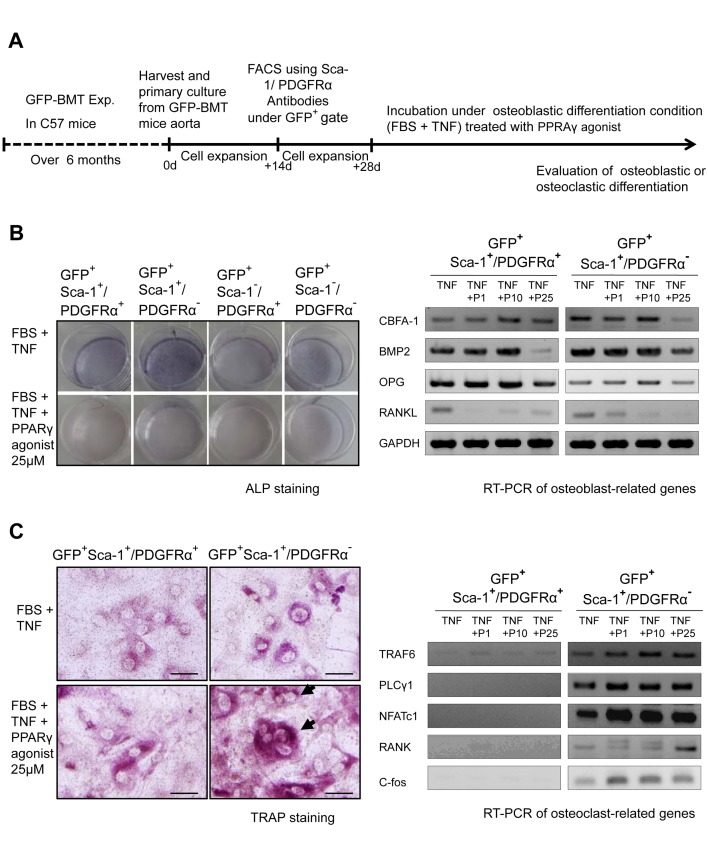
Ex vivo BM-derived vascular calcifying progenitor cells modulated by PPARγ activation toward osteoblastic/osteoclastic differentiations. (A) Experimental outline (*n* = 10, performed in triplicate). (B and C) BM-derived GFP^+^ cells were treated with rosiglitazone under osteoblastic differentiation conditions (10% FBS+10 ng/ml TNF-α). ALP staining (B) indicated that PPARγ activation suppressed osteoblastic differentiation. TRAP staining (C) and RT–PCR revealed that PPARγ activation induced the osteoclastic differentiation of Sca-1^+^/PDGFRα^−^ cells. P, PPARγ agonist 1, 10, or 25 µM. Bars: 100 µm.

PPARγ activation has been suggested to repress osteoblastogenesis and to activate osteoclastogenesis [16],[23]. These observations led us to hypothesize that PPARγ activation in calcifying progenitor cells might reverse the process of VC. We first confirmed endogenous PPARγ expression in Sca-1^+^/PDGFRα^+^ and Sca-1^+^/PDGFRα^−^ calcifying progenitor cells (Figure S8A). BM-derived vessel-resident GFP^+^ cells then were cultured in FBS and TNF-α to strongly induce osteoblast differentiation and the influence of an added PPARγ agonist was assessed (Figure 5A). In the absence of PPARγ activation, osteoblastic differentiation was markedly induced (Figure 5B). Under the same conditions, PPARγ activation of Sca-1^+^/PDGFRα^−^ cells suppressed osteoblastogenesis (Figure 5B) and promoted osteoclastic differentiation (Figure 5C). PPARγ activation suppressed the expression of osteoblast-related genes and enhanced the expression of osteoclast-related genes, facilitating the emergence of TRAP-positive cells (Figure 5B,C) as confirmed by real-time PCR (Figure S8B,C). These results indicated that PPARγ activation not only suppressed the osteoblastic differentiation of vascular cells, but also promoted the osteoclastic differentiation of bidirectional calcifying progenitor cells. Hence, the detrimental process of VC may be preventable as well as reversible.

### Calcifying Progenitor Cells Regulate in Vivo Ectopic Mineralization

**Figure 6:**
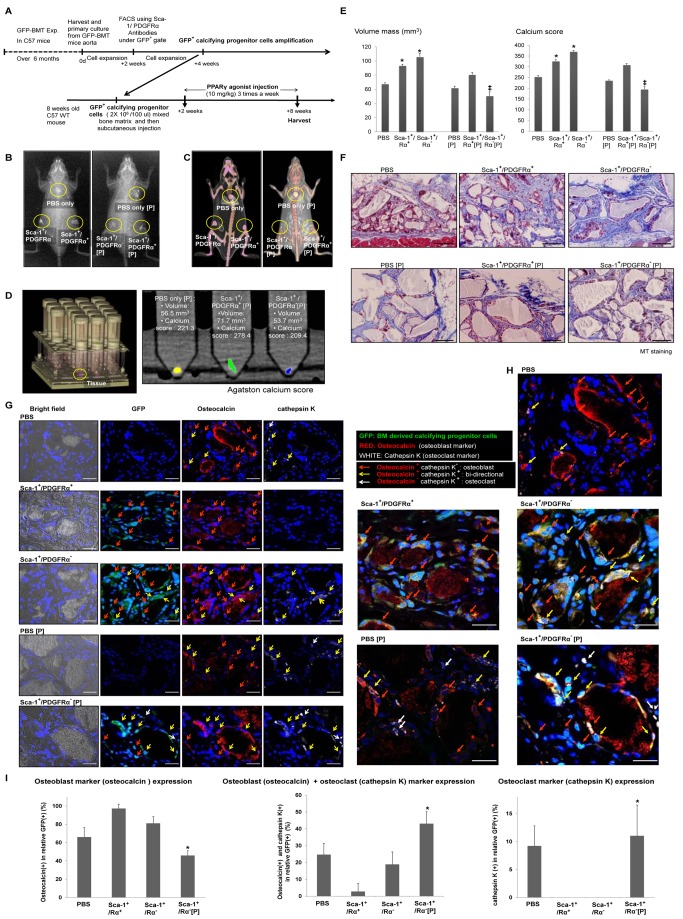
Calcifying progenitor cells in an in vivo bone-like structure matrix calcification model. (A) Timeline of the calcification model (*n* = 10 per group). (B) X-ray and (C) CT of GFP^+^Sca-1^+^ cells mixed with bone matrix and implanted subcutaneously into C57 mice. (D) Quantification of CT results using Agatston scoring. (E) Volume mass and calcium scores of mice injected with GFP^+^Sca-1^+^ cells with or without concurrent PPARγ agonist treatment. *P<0.005 versus PBS-treated mice. ‡*P*<0.001 versus mice injected with GFP^+^Sca-1^+^/PDGFRα^−^ cells. (F) MT staining. Bars: 100 µm. (G) Immunostaining with osteocalcin, an osteoblastic marker, and cathepsin K, an osteoclastic marker, to determine the differentiation fate and the effect of PPARγ activation on injected Sca-1^+^ cells. Bars: 20 µm. (H) Enlarged immunostaining images showing osteocalcin and cathepsin K. Bars: 20 µm. (I) Osteoblastic, osteoclastic, and bidirectional cell counts. GFP^+^Sca-1^+^/PDGFRα^+^ cells primarily expressed osteoblast markers. Most of the GFP^+^Sca-1^+^/PDGFRα^−^ cells differentiated into osteoblast-like cells, but some cells differentiated into bidirectional cells. When PPARγ was activated, GFP^+^Sca-1^+^/PDGFRα^−^ cells differentiated more frequently into bidirectional cells or osteoclasts (*n* = 10 per group). *P<0.005 compared to mice injected with GFP^+^Sca-1^+^/PDGFRα^−^ cells. P, PPARγ agonist.

To verify the in vivo calcifying ability of progenitor cells and the efficacy of PPARγ activation, we isolated GFP^+^ calcifying progenitor cells from the arteries of C57 mice that underwent GFP–BMT. GFP^+^ progenitor cells were combined with bone matrix, implanted subcutaneously into WT C57 mice, and a PPARγ agonist was injected (Figure 6A). After 8 wk of PPARγ agonist treatment, X-ray and three-dimensional computed tomography (CT) of the implanted mice indicated a higher mineralization density of a bone-like structure in the Sca-1^+^ cell groups compared with mice administered phosphate-buffered saline (PBS). This mineralization of mass was remarkably inhibited in Sca-1^+^ cells treated with a PPARγ agonist compared with cells without PPARγ activation. Specifically, the bone-like structure of Sca-1^+^/PDGFRα^−^ cells was dramatically reduced by PPARγ activation (Figure 6B,C). The volume and calcium scores of Sca-1^+^ cells were higher than those of either PBS-treated or Sca-1^−^ cells quantified using the Agatston method (Figure 6D) [24]. These scores were significantly increased in Sca-1^+^/PDGFRα^−^ cells treated with a PPARγ agonist. However, no difference was detected between mice injected with PBS or PBS and a PPARγ agonist (Figure 6E). Masson's trichrome (MT) staining indicated that Sca-1^+^ cell injection enhanced blue staining, indicating bone-like tissues, whereas PPARγ treatment decreased blue staining (Figure 6F).

Therefore, we sought to determine the effect of PPARγ activation in Sca-1^+^ cells by double-staining with osteocalcin and cathepsin K. Injected GFP^+^Sca-1^+^/PDGFRα^+^ cells were osteocalcin-positive and cathepsin K-negative. Injected GFP^+^Sca-1^+^/PDGFRα^−^ cells were primarily osteocalcin-positive, but some GFP^+^Sca-1^+^/PDGFRα^−^ cells were doubly positive for osteocalcin and cathepsin K. Exclusively cathepsin K-positive cells were observed only in the mass of GFP^+^Sca-1^+^/PDGFRα^−^ cells treated with a PPARγ agonist (Figure 6G–I). These results demonstrate that BM-derived Sca-1^+^/PDGFRα^−^ cells are bidirectional, and PPARγ may act as a regulator of VC in these cells.

### Bidirectional Calcifying Progenitor Cells Regulate Atherosclerotic Calcification in Apoe^−/−^ Mice

**Figure 7:**
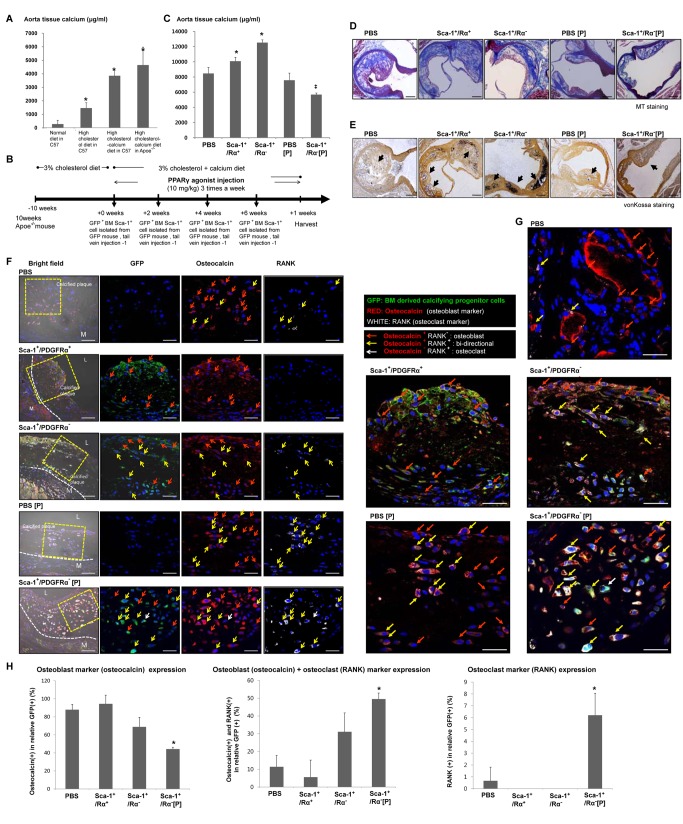
Calcifying progenitor cells induce calcification in atherosclerotic plaques of Apoe^−/−^ mice. (A) Calcium accumulation levels in mouse arteries according to diet. *P<0.005 versus C57 fed a normal diet (*n* = 10 per group). (B) Experimental timeline. (C) Calcium accumulation levels in the aortas of Apoe^−/−^ mice according to BM cell type injected and PPARγ activation. *P<0.001 versus PBS-treated group. ‡*P*<0.001 versus mice injected with BM Sca-1^+^/PDGFRα^−^ cells. (D) Atherosclerotic plaque with MT staining. Bars: 200 µm. (E) Calcification induction in atherosclerotic plaques was detected by von Kossa staining. Arrows indicate calcified areas. Bars: 200 µm. (F) Atherosclerotic calcification plaque immunostained with osteocalcin, an osteoblastic marker, and RANK, an osteoclastic marker, to determine the fates of infiltrating BM-derived calcifying progenitor cells in the presence/absence of PPARγ activation. Bars: 20 µm. (G) Image enlargement showing osteocalcin and RANK immunostaining. Bars: 20 µm. (H) Osteoblastic, osteoclastic, and bidirectional cell counts in the artery. GFP^+^Sca-1^+^/PDGFRα^+^ cells primarily expressed osteoblast markers. GFP^+^Sca-1^+^/PDGFRα^−^ cells mainly differentiated into osteoblast-like cells, but some infiltrating cells differentiated into bidirectional cells. When PPARγ was activated, differentiating GFP^+^Sca-1^+^/PDGFRα^−^ cells shifted from osteoblast-like to bidirectional cells or osteoclasts (*n* = 10 per group). *P<0.005 compared to mice injected with BM-derived Sca-1^+^/PDGFRα^−^ cells. P, PPARγ agonist.

We assessed the accumulation of aortic calcium in response to diet. Mice fed a high cholesterol/calcium diet showed a significantly higher level of calcium accumulation than mice fed a normal diet (Figure 7A). To understand the function of calcifying progenitor cells in calcified atherosclerotic plaques, we isolated BM cells from a GFP mouse. BM-GFP^+^Sca-1^+^/PDGFRα^+^ and GFP^+^Sca-1^+^/PDGFRα^−^ cells were injected with or without a PPARγ agonist into the tail veins of Apoe^−/−^ mice every 2 wk over a period of 8 wk (Figure 7B). The cell properties of injected BM cells, BM-GFP^+^Sca-1^+^/PDGFRα^+^ cells, and GFP^+^Sca-1^+^/PDGFRα^−^ cells were compared between normal (Figure S7) and high-cholesterol (Figure S9) diets, and no differences were identified.

The calcium accumulation levels of each cell group were measured from harvested arteries. The groups injected with BM-GFP^+^Sca-1^+^ cells, especially BM-GFP^+^Sca-1^+^/PDGFRα^−^ cells, showed greater calcium accumulation in tissues than animals injected with PBS. This calcium accumulation was significantly avoided by concurrent treatment with a PPARγ agonist. However, no difference in calcium accumulation was measured between mice injected with PBS versus PBS and a PPARγ agonist. Therefore, the preventative effects of the GFP^+^Sca-1^+^/PDGFRα^−^ + PPARγ condition should be mainly attributed to the BM-GFP^+^Sca-1^+^/PDGFRα^−^ cells, not the PPARγ agonist (Figure 7C). Mice injected with GFP^+^Sca-1^+^/PDGFRα^−^ cells showed significantly more severe atherosclerotic calcified plaques identified by MT and von Kossa staining. These plaques were prevented by the addition of GFP^+^Sca-1^+^/PDGFRα^−^ cells and a PPARγ agonist (Figure 7D,E).

Additional studies were conducted to determine the characteristics of the injected GFP^+^Sca-1^+^ cells that infiltrated the calcified atherosclerotic plaques. GFP^+^ cells were double-stained with osteocalcin and RANK. In the calcified atherosclerotic plaques of PBS injected mice, osteocalcin-positive osteoblasts and a few RANK^+^ osteoclasts were observed. Mice injected with GFP^+^Sca-1^+^/PDGFRα^+^ cells harbored plaques infiltrated primarily by osteocalcin-positive cells. Mice injected with GFP^+^Sca-1^+^/PDGFRα^−^ cells contained plaques that were infiltrated with both osteocalcin and RANK double-positive cells as well as osteocalcin-positive osteoblasts. These data suggest that the Sca-1^+^/PDGFRα^−^ cells we observed in calcified atherosclerotic plaques possessed both osteoblastic and osteoclastic differentiation potentials. PPARγ agonist treatment significantly decreased the infiltration of osteoblasts and increased the infiltration of osteoclast or double-positive cells into the plaques (Figure 7F–H). We conclude that PPARγ activation can decrease atherosclerotic calcification by modulating the fate of bidirectional Sca-1^+^/PDGFRα^−^ cells.

## Discussion

**Figure 8:**
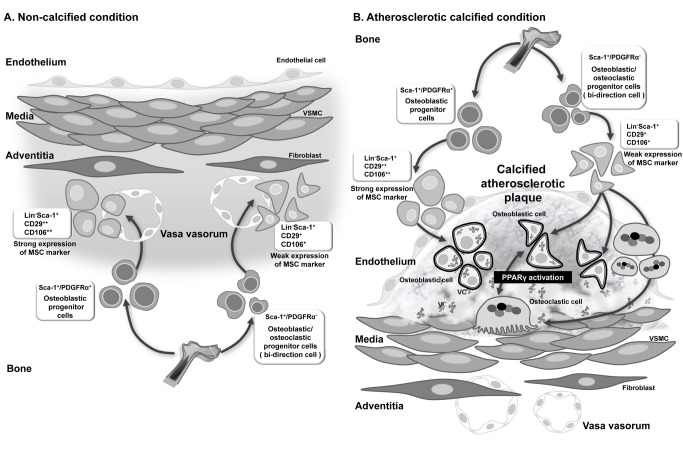
The regulation of vascular calcification by calcifying progenitor cells. Illustration of calcifying/decalcifying progenitor cells and their proposed roles in noncalcified conditions (A) and atherosclerotic plaques (B). Sca-1^+^ progenitor cells do not express hematopoietic lineage markers but express MSC-like markers. Sca-1^+^/PDGFRα^−^ progenitor cells differentiate into osteoblasts/osteoclasts bidirectionally, whereas Sca-1^+^/PDGFRα^+^ progenitor cells are committed to osteoblastic fates. PPARγ activation can shift the direction of differentiation of Sca-1^+^/PDGFRα^−^ progenitor cells toward osteoclasts, but it cannot change the differentiation of Sca-1^+^/PDGFRα^+^ progenitors. (A) Under homeostatic noncalcified conditions, circulating BM-derived calcifying cells (Sca-1^+^ cells) infiltrated the adventitia through the vasa vasorum. (B) Under pathologic atherosclerotic calcification or therapeutic intravascular cell delivery, these cells might infiltrate into the intima directly from the blood.

VC occurs by an actively regulated process, but the origin of vascular calcifying cells has not been established. In this study, we demonstrated the origin, in vitro and ex vivo characteristics, and differentiation potentials of a population of vascular calcifying progenitor cells. These cells were confirmed to modulate calcification/decalcification through a series of in vivo experiments. A schematic illustration of calcifying/decalcifying progenitor cells and their proposed actions is presented in Figure 8.

Recent studies have shown that circulating BM-derived cells could infiltrate via the vasa vasorum (micro vessel) [25]. However, it remained unclear how cells could infiltrate the intima. Others have suggested that the vasa vasorum is increased in the adventitia during inflammation. The vasa vasorum may function as a hallway for cell migration into the adventitia toward atherosclerotic plaques [26],[27]. However, circulating BM-derived cells could directly infiltrate the atherosclerotic plaques. Shimizu et al. demonstrated that cells infiltrating the intima originated as circulating BM-derived cells [28],[29]. Cho et al. reported that cells infiltrating the adventitia did not migrate into the intima and only circulating cells infiltrated the plaque lesion [30]. Our results suggest that under homeostatic noncalcified conditions, circulating BM-derived calcifying cells (Sca-1^+^ cells) infiltrated the adventitia through the vasa vasorum. However, under pathologic atherosclerotic calcification or therapeutic intravascular cell delivery, these cells might infiltrate into the intima directly from the blood.

Osteoblasts originate from MSCs [6], whereas osteoclasts are cell–cell fused multinucleated cells derived from granulocyte/macrophage hematopoietic progenitor cells in the bone [31]. Recently, Eghbali-Fatourechi et al. reported that circulating osteoblastic cells are elevated in adolescents and in patients with fractures, reflecting increased bone formation [32]. However, the roles of circulating osteoblastic cells during VC have not been elucidated. Osteoclast-like cells in the vasculature may originate from circulating hematopoietic cells that were recruited to blood vessels [33], but this possibility has not been confirmed. Here, we demonstrate that a subtype of calcifying progenitor cells, Sca-1^+^/PDGFRα^−^ cells, originate in BM and infiltrate arteries. Sca-1^+^/PDGFRα^−^ cells are mesenchymal and possess bidirectional osteoblastic/osteoclastic potentials. The bidirectionality of Sca-1^+^/PDGFRα^−^ cells was supported by Masuda et al. (2001), who identified ALP and TRAP double-positive cells in tissues during endochondral ossification [34]. Others have described the mesenchymal characteristics of CD45^−^TER119^−^Sca-1^+^/PDGFRα^+^ cells and Sca-1^+^/PDGFRα^−^ cells, and injected Sca-1^+^/PDGFRα^+^ cells primarily differentiate into osteoblasts in vivo [35].

We examined the in vivo modulation of calcifying progenitor cell differentiation by PPARγ activation using an ectopic model and an atherosclerotic calcification model. When tissue was double-stained with osteoblast (osteocalcin) and osteoclast (cathepsin K or RANK) markers, Sca-1^+^/PDGFRα^−^ cells mainly differentiated into osteocalcin-positive cells. However, when cells were cultured with a PPARγ agonist, their differentiation shifted from osteoblastic to osteoclastic or double-positive cells in tissue [16],[36]. These results suggest that PPARγ activation shifts Sca-1^+^/PDGFRα^−^ cells from osteoblastic to osteoclastic differentiation in vivo [37],[38]. The concentration of the PPARγ agonist, rosiglitazone (5 mg/kg/d), used in the mice study was higher than the human AUC (area under curve) of the FDA-recommended clinical daily dose. Thus, additional study is warranted to find an efficacious novel PPARγ agonist. We also investigated the mechanism by which PPARγ facilitates osteoblastic differentiation. PPARγ activation suppresses osteoblastogenesis by favoring adipogenesis and improving osteoclastogenesis [23],[39]. Wei et al. reported that the transcriptional co-activators of PPARγ, PGC1β and ERRα, enable cell differentiation into osteoclasts and adipocytes [40]. These researchers demonstrated that PPARγ activation upregulated osteoclast differentiation by inducing GATA2, which is required to generate osteoclast progenitors, and by activating PPARγ ligand via c-fos induction, thereby stimulating osteoclast differentiation [40],[41]. Sca-1^+^/PDGFRα^−^ cells cultured in TNF-α with PPARγ activation suppressed osteoblastogenesis and enhanced the expression levels of osteoclast-related genes, especially c-fos expression, compared with cells in the absence of PPARγ stimulation.

Sca-1^+^/PDGFRα^−^ cells displayed both osteoblastic and osteoclastic differentiation potentials in this study. However, the hierarchy between Sca-1^+^/PDGFRα^+^ and Sca-1^+^/PDGFRα^−^ cells remains unclear. Sca-1^+^/PDGFRα^−^ likely are hierarchically above Sca-1^+^/PDGFRα^+^ cells because the former are capable of differentiating into osteoblasts or osteoclast-like cells, whereas the latter differentiate into osteoblasts only. Sca-1^+^/PDGFRα^−^ cells have multi-potentiality with relatively weak MSC marker expression and scanty hematopoietic marker expression. These cells can differentiate into osteoclast-like cells that usually are derived from hematopoietic stem cells, implying that they might be positioned hierarchically between hematopoietic stem cells and MSCs. When they are committed into a more specific lineage, Sca-1^+^/PDGFRα^−^ cells probably lose their bidirectional potential, becoming unidirectional Sca-1^+^/PDGFRα^+^ cells. Notably, calcifying progenitor cells in the artery and BM are not abundant.

The present study has several limitations. We have characterized calcifying progenitor cells in C57 wild-type mice, but not in C57 background Apoe^−/−^ mice. Thus, those cells may be different or behave differently when added to atherosclerotic environment. The numbers of Sca-1^+^/PDGFRα^−^ cells in the BM were less than 1% of the total BM cells. However, the infiltration of Sca-1^+^/PDGFRα^−^ cells into the vessels was associated with marked aggravation of atherosclerotic calcification. Based on the percentage of cells measured by flow cytometry [42], BM-derived Sca-1^+^/PDGFRα^−^ osteocalcin-positive cells comprised 2% of the total cells infiltrating the artery, and Sca-1^+^/PDGFRα^−^ osteocalcin^+^ RANK^+^ (or cathepsin K^+^) cells comprised 1%. Under PPARγ-activated conditions, the number of Sca-1^+^/PDGFRα^−^ osteocalcin^+^ RANK^+^ (or cathepsin K^+^) cells increased by 2%, and Sca-1^+^/PDGFRα^−^ RANK^+^ cells increased by 0.5%. These cells are expected to substantially impact VC homeostasis even if their absolute numbers are low. It is obvious that VSMCs play an important role in calcification of the vasculature [43],[44]. An analysis of calcification induction in these cells (Sca-1^−^/PDGFRα^+^) is beyond the scope of the present study. Our focus is on the characteristics and significance of Sca-1^+^/PDGFRα^+^ cells and Sca-1^+^/PDGFRα^−^ cells.

In conclusion, our data demonstrate that BM-derived, MSC-like Sca-1^+^/PDGFRα^−^ cells reside in the arterial adventitia and possess differentiation potentials toward both osteoblastic and osteoclastic lineages. Even under calcifying conditions, PPARγ activation promoted osteoclastic differentiation of bidirectional cells, both in vitro and ex vivo. Finally, we confirmed the in vivo relevance of bidirectional progenitor cells that are modulated by PPARγ activation. This subtype of BM-derived circulating and vessel-resident calcifying progenitor cells offers a new therapeutic target for VC. PPARγ activation in these cells has the potential for VC management.

## Materials and Methods

### Animals

Wild-type C57BL/6J mice (KBT Oriental Co Ltd., Charles River Grade, Tosu, Saga, Japan) and ubiquitous enhanced GFP-expressing transgenic mice with a C57 background (The Jackson Laboratory, Bar Harbor, Maine, USA) were used in this study. Apoe^−/−^ (B6.129P2-Apoetm1Unc/J; The Jackson Laboratory) mice were used as a model of atherosclerotic calcification. All mice used in this study were males [45]. All procedures were performed in accordance with the Institutional Animal Care and Use Committee of Seoul National University Hospital.

### Cell Isolation and Culture

Primary cells cultured from aorta, including the media and adventitia, were prepared by enzyme digestion using collagenase type II (GIBCO) as previously described [46]. Cells were cultured in Mesencult media (Stem Cell Technologies). To examine calcifying progenitor cells, cells were cultured for 2 wk and harvested. They then were stained with the surface markers, Sca-1 (BD Pharmingen) and PDGFRα (Cell Signaling). Sorting was performed using a FACSAria (Becton Dickinson). Sorted cells were divided into the following four groups: Sca-1^+^/PDGFRα^+^, Sca-1^+^/PDGFRα^−^, Sca-1^−^/PDGFRα^+^, and Sca-1^−^/PDGFRα^−^. To confirm the characteristics of calcifying progenitor cells, cells were stained with using a hematopoietic lineage antibody cocktail (Lin^−^) containing antibodies targeting CD3, CD11b (monophage/macrophage marker), CD45R/B220, TER-11, and Ly-6G (BD Pharmingen) or with antibodies targeting CD29 or CD106 (R&D Systems).

### Osteoblastic Differentiation

Sorted cells were cultured for 14 d under three osteoblastic differentiation culture conditions in MEM alpha media (GIBCO): 10% charcoal-stripped FBS only, 10% charcoal-stripped FBS with 10 ng/ml TNF-α [15], and 10% charcoal-stripped FBS containing 6 mM CaCl_2_, 10 mM sodium pyruvate, and 10 mM β-glycerophosphate [47]. Cells were harvested on days 1, 3, 7, and 14 for reverse transcriptase (RT)–PCR or real-time PCR, ALP staining, and ALP activity. To measure ALP activities, cells were grown under osteoblastic differentiation conditions. Harvested cells were washed twice with PBS, lysed in 0.05% Triton-X100 in PBS, and subjected to three freeze/thaw cycles. Cell lysate supernatants were transferred to 96-well plates and were incubated with 50 µl alkaline buffer (Sigma) for 10 min and 50 µl phosphatase substrate capsules (Sigma) until yellow color was observed. P-nitrophenol standard solution (Sigma) was used to generate a standard curve. Absorbance at 405 nm was measured using a plate reader (Bio-Rad). ALP activities were normalized to the protein concentrations of the samples. ALP staining was performed using the BCIP/NBT substrate system (Dako). To evaluate gene expression under osteoblastic differentiation conditions, RT–PCR was performed as previously described [48]. Complementary DNA was PCR-amplified using the osteoblastic differentiation markers CBFA-1, OPG, and RANKL (Tables S1 and S2) [49].

### Osteoclastic Differentiation

Sorted cells were cultured in MEM alpha medium with 10% charcoal-stripped FBS (GIBCO), 10 ng/ml M-CSF (R&D Systems), and 100 ng/ml RANKL (R&D Systems) for 7 d [17]. Cells were harvested weekly for RT–PCR. After 7 d of osteoclast differentiation induction, TRAP staining (Sigma) was performed as a measure of osteoclastic activities. TRAP-positive multinucleated cells (>3 nuclei) were counted. RT–PCR or real-time PCR was performed to amplify the following osteoclastic differentiation markers: nuclear factor of activated T-cells-1 (NFATc1), phospholipase C, gamma-1 (PLCγ1), TNF receptor-associated factor-6 (TRAF6), RANK, RANKL, c-Fos [50], and GAPDH (Table S1 and S2). Sca-1^+^/PDGFRα^+^ and Sca-1^+^/PDGFRα^−^ cells were seeded onto calcium phosphate-coated discs (BD bioscience) in 12-well culture plates and were cultured in osteoclastic differentiation media. After 21 d, cells were removed from the discs using a bleach solution. The discs were washed three times with distilled water, air dried, and examined by SEM [51]. Alternatively, after 21 d, cells were stained with FITC-conjugated phalloidin (Sigma) and observed by confocal microscopy [52].

### Clonal Expansion and Osteogenic, Chondrogenic, and Adipogenic Differentiation

To detect MSC-like properties of vessel-resident calcifying progenitor cells, the cells were induced to differentiate into osteoblasts, adipocytes, or chondrocytes by varying the culture conditions. The osteoblast differentiation media consisted of 10% charcoal-stripped FBS with 6 mM CaCl_2_, 10 mM sodium pyruvate, and 10 mM β-glycerophosphate [47]. After 14 d, osteoblastic cells were stained with Alizarin Red S (Sigma). After 28 d, differentiated cells, cultured in adipocyte differentiation media (Stem Cell Technologies) or chondrocyte differentiation media (R&D Systems), were stained with Oil Red O (Sigma) or Safranin O solution (Sigma), respectively. To detect single clonal expansion, sorted arterial cells from C57 mice were seeded into 6-well plates (1×10^4^ cells/well) or into 96-well plates (1 cell/well). After 14 d of culture, colonies were Giemsa-stained (Sigma). To examine whether calcifying progenitor cells were derived from single cells that could differentiate into osteoblasts or osteoclasts, cells were seeded into 12-well plates (200 cells/well) and were cultured for 7 d. Cells then were induced to differentiate into osteoblasts or osteoclasts during the following 14 d. Differentiated cells were stained with Alizarin Red S or TRAP and measured the mRNA expression of the following Adipocyte and chondrocyte differentiation markers: C/EBP (CCAAT-enhancer-binding proteins) α and β, PPARγ [53], collagen 1a1, 2a1, aggrecan [54], and GAPDH (Table S3).

### Measurement of Aortic Tissue Calcium

To measure calcium levels in aortas of mice fed a normal diet, a high cholesterol diet, or a high cholesterol/calcium diet (7% fat, 3% cholesterol, 200,000 IE/kg vitamin D, 3,000 mg/kg calcium, 1.700 mg/kg phosphate). Isolated aorta tissues were dried at 70°C overnight and weighed. Dried tissues were dissolved in 2 M HCl at 70°C for 24 h. Pellets were obtained and HCl was removed using a vacuum dryer. Dried pellets then were added to 1 ml distilled water. Calcium and phosphorous levels were measured relative to total protein using an autoanalyzer (Hitachi 7070, Tokyo, Japan).

### In Vivo Ectopic Calcification Assay

Calcifying progenitor cells were collected and sorted from the arteries of GFP–BMT mice that had been fed a high cholesterol diet. Cells were mixed with triosite (Zimmer) and were incubated for 1 h. Transplanted cells then were mixed with fibrinogen and thrombin before being implanted into a subcutaneous pocket of an 8-wk-old C57 mouse. Eight weeks later, ectopic mineralization was assessed by X-ray (TU-3000DR, Hitachi) and CT (Somatom Definition; Siemens Medical Solutions, Forchheim, Germany) and was quantified using the Agatston score [32],[55],[56]. Ectopically mineralized tissues were harvested and embedded in paraffin.

### Atherosclerotic Calcification in Apoe^−/−^ Mice

BM-derived cells were isolated and sorted from GFP mice. BM-GFP^+^Sca-1^+^/PDGFRα^+^ and GFP^+^Sca-1^+^/PDGFRα^−^ cells (1×10^6^/100 µl) were injected into the tail veins of Apoe^−/−^ mice 4 times at 2-wk intervals. Apoe^−/−^ mice were fed a high cholesterol diet for 10 wk prior to cell injection. Mice continued the high cholesterol/calcium diet for another 8 wk following injection. To test the effect of PPARγ activation in vivo, the PPARγ agonist (rosiglitazone, 10 mg/kg) was injected intraperitoneally into mice 3 times per week for 8 wk. Arteries were harvested, and calcium and phosphorus levels were measured. Atherosclerotic plaque formation and calcium deposits were evaluated by MT and von Kossa staining.

### Statistical Analysis

All data were presented as means ± SEM. Intergroup comparisons were performed using the Student's *t* test or one-way analysis of variance (ANOVA). Data obtained at different time points were analyzed by repeated measures ANOVA. SPSS v16.0 was used for all statistical analyses, and *P*<0.05 was considered statistically significant.

## Supporting Information

Figure S1 Various markers of stem/progenitor cell expression in vasculature. Various markers of stem/progenitor cells and mature blood cells were applied to identify the vascular calcifying progenitor cells in aortas. C-kit, CD34, and AC133-positive cells were relatively scarce, and the majority of adventitial cells did not express these markers (*n* = 5). Green, c-kit; green, CD 45; red, CD34; red, AC133C. The number 1 and 2 images are higher magnification than the left panel. The number 1 and 2 indicate adventitia and media, respectively. The white dashed lines indicate the media. A, Adventitia; L, Lumen; blue, Sytox blue for nuclei. Bars: yellow, −100 µm; white, −20 µm.
(TIF)

Figure S2 Isolation and sorting of calcifying progenitor cells from the mouse artery 2 wk after cell expansion. (A) A schematic representation of calcifying progenitor cell isolation experiments (*n* = 10). (B) FACS gate using Sca-1 and PDGFRα antibodies staining. (C) The purities of the isolated four cell groups were confirmed by immunofluorescent staining. Bars: 50 µm.
(TIF)

Figure S3 Isolation and sorting of calcifying progenitor cells from the mouse artery immediately after single cell suspension. (A) A schematic representation of calcifying progenitor cell isolation experiments (*n* = 10). (B) Sorting gate using Sca-1/PDGFRα staining. (C) Under osteoblastic differentiation conditions containing the 10% FBS+1.25 mM CaCl_2_+2 mM β-glycerolphosphate, the numbers of ALP staining positive cells are significantly higher in Sca-1+ cells (Sca-1^+^/PDGFRα^+^ and Sca-1^+^/PDGFRα^−^ cells) than Sca-1− cells (Sca-1^−^/PDGFRα^+^ and Sca-1^−^/PDGFRα^−^ cells). (D) Under osteoclastic differentiation conditions, Sca-1^+^/PDGFRα^−^ cells differentiated into TRAP positive, multinucleated cells on differentiation day 7. Bars: 100 µm.
(TIF)

Figure S4 Osteoblastic or osteoclastic differentiation-related gene expression of the calcifying progenitor cells in the vasculature. Three different conditions of osteoblast induction were examined, 10% FBS only, 10% FBS+10 ng/ml TNF-α, and 10% FBS+1.25 mM CaCl_2_+2 mM β-glycerolphosphate. (A) Under osteoblastic conditions, Sca-1^+^ cells showed higher expression levels of osteoblast-related genes, than Sca-1^−^ cells on differentiation day 7. *P<0.05 versus Sca-1^−^/PDGFRα^−^ cells. (B) Under moderate osteoblastic induction conditions (FBS only), the osteoblastic related gene expression levels were lower than potent osteoblastic differentiation (FBS+TNF-α or FBS+CaCl_2_) on differentiation day 7. *P<0.05 versus FBS only culture condition. (C) Under osteoclastic differentiation conditions, the osteoclast-related genes were increased in Sca-1^+^/PDGFRα^−^ cells (performed in triplicate) on differentiation day 14. *P<0.05 versus Sca-1^+^/PDGFRα^+^ cells.
(TIF)

Figure S5 Fates of Sca-1^+^/PDGFRα^−^ cells cultured in mixed medium containing osteoblastic and osteoclastic stimulators. To test the fates of Sca-1^+^/PDGFRα^+^ and Sca-1^+^/PDGFRα^−^ cells, these cells cultured in mixed medium containing FBS and TNF-α (osteoblastic differentiation stimulators) with RANKL and M-CSF (osteoclastic differentiation stimulators). Cells were stained for osteocalcin, an osteoblast marker, and cathepsin K, an osteoclast marker. Seven days after incubation in mixed medium, both Sca-1^+^/PDGFRα^+^ and Sca-1^+^/PDGFRα^−^ cells dominantly expressed osteocalcin. Interestingly, a few Sca-1^+^/PDGFRα^−^ cells expressed cathepsin K but did not form multinucleated cells. Sca-1^+^/PDGFRα^+^ cells did not express cathepsin K. Bars: 50 µm.
(TIF)

Figure S6 The role of PDGFRα in osteoblastic differentiation. Sca-1^+^/PDGFRα^+^ cells treated with/without 10 ng/ml or 25 ng/ml PDGFBB in charcoal-stripped, 10% FBS-supplemented MEM alpha medium for 0, 3, 7, or 14 d. (A) After 7 d, Osteoblast-related gene expression, CBFA-1, ostrix, and OPG were unregulated by PDGFBB treatment. *P<0.05 versus FBS only treated group. (B) ALP expression in cells was not affected by the presence/absence of PDGFBB as evaluated by FACS. TNF-α-treated cells were used as a positive stimulation control.
(TIF)

Figure S7 Ex vivo osteoblastic and osteoclastic differentiation of BM-derived vascular calcifying progenitor cells. (A) Experimental outline describing GFP^+^ calcifying progenitor cell isolation and osteoblastic/osteoclastic differentiation. Experiments were performed in triplicate. (B) The purities of the isolated GFP^+^ calcifying progenitor cell groups were confirmed by immunofluorescent staining. Bars: 50 µm. Under the three different osteoblastic differentiation conditions, (C) ALP staining and (D) ALP activity determinations showed that BM-derived Sca-1^+^/PDGFRα^−^ cells possessed the greatest osteoblastic differentiation potential, followed by Sca-1^+^/PDGFRα^+^ cells. Experiments were performed in triplicate. *P<0.01 versus Sca-1^−^/PDGFRα^+^ cells. (E) Under osteoclastic differentiation conditions, only Sca-1+/PDGFRα− cells differentiated into multinucleated cells (>3 nuclei) and expressed osteoclast-related genes (counting field *n* = 5 per group). Bars: 100 µm (B and C). G, GFP; S, Sca-1; Rα, PDGFRα. *P<0.001 versus Sca-1^−^/PDGFRα^+^ cells. Bars: 100 µm.
(TIF)

Figure S8 A PPARγ agonist modulates the gene expression of *ex vivo* BM-derived vascular calcifying progenitor cells under osteoblastic or osteoclastic differentiation conditions. (A) Sca-1^+^ (Sca-1^+^/PDGFRα^+^ and Sca-1^+^/PDGFRα^−^) and Sca-1^−^ (Sca-1^−^/PDGFRα^+^ and Sca-1^−^/PDGFR^−^) cells expressed endogenous PPARγ in the absence of a PPARγ agonist. *P*>0.05 versus Sca-1^−^/PDGFRα^−^ cells. (B) Under osteoblastic differentiation conditions (FBS+TNF-α) and in the presence of rosiglitazone for 5 d GFP^+^Sca-1^+^/PDGFRα^+^ cells suppressed osteoblast-related genes, OPG, CBFA-1, and ALP and did not express osteoclast-related genes, RANK and TRAF6. *P<0.05 versus GFP^+^Sca-1^+^/PDGFRα^+^ cells treated TNF-α without PPARγ agonist. (C) BM-derived GFP^+^Sca-1^+^/PDGFRα^−^ cells suppressed osteoblast-related genes and promoted osteoclast-related genes. *P<0.05 versus GFP^+^Sca-1^+^/PDGFRα^+^ cells treated TNF-α without PPARγ agonist. P, PPARγ agonist 1, 10, or 25 µM.
(TIF)

Figure S9 Ex vivo osteoblastic and osteoclastic differentiation of BM-derived vascular calcifying progenitor cells in mice fed a high cholesterol diet. (A) An outline of experiments for osteoblastic and osteoclastic differentiation. (B) The purities of the isolated GFP^+^ calcifying progenitor cell groups were confirmed by immunofluorescent staining. Bars: 50 µm. (C) Under the three osteoblastic differentiation conditions, ALP staining and RT-PCR analysis indicated that BM-derived Sca-1^+^/PDGFRα^−^ cells possessed the greatest osteoblastic differentiation potential followed by Sca-1^+^/PDGFRα^+^ cells. Experiments were performed in triplicate. (D) Under osteoclastic differentiation conditions, only Sca-1^+^/PDGFRα^−^ cells differentiated into multinucleated cells (>3 nuclei) and expressed osteoclast-related genes (counting field *n* = 5 per group). Bars: 100 µm (B and C). G, GFP; S, Sca-1; Rα, PDGFRα. *P<0.001 versus Sca-1^−^/PDGFRα^+^ cells. Bars: 100 µm.
(TIF)

Table S1 Primer sequences of RT-PCR, TMs, and the sizes of PCR products.
(DOCX)

Table S2 Primer sequences for osteoblast and osteoclast marker of real-time PCR.
(DOCX)

Table S3 Primer sequences for adipocyte and chondrocyte marker of real-time PCR.
(DOCX)

## Abbreviations

ALP: alkaline phosphatase
BM: bone marrow
BMT: bone marrow transplantation
MSCs: mesenchymal stem cells
NFACT1: nuclear factor of activated T-cells
OPG: osteoprotegerin
PDGFRα: platelet-derived growth factor receptor alpha
PLCγ1: phospholipase c, gamma-1
PPARγ: peroxisome proliferator activated receptor-gamma
RANK: receptor activator for nuclear factor κB
RANKL: receptor activator for nuclear factor κB ligand
Sca-1: stem cell antigen-1
TRAF6: TNF receptor associated factor 6
TRAP: tartrate-resistant acid phosphatase
VC: vascular calcification
