# Supplementary figures and images for: Vascular Calcifying Progenitor Cells Possess Bidirectional Differentiation Potentials
Source: PLoS Biol. 2013 Apr 9;11(4):e1001534. doi: 10.1371/journal.pbio.1001534 (PMC3621676; doi:10.1371/journal.pbio.1001534)

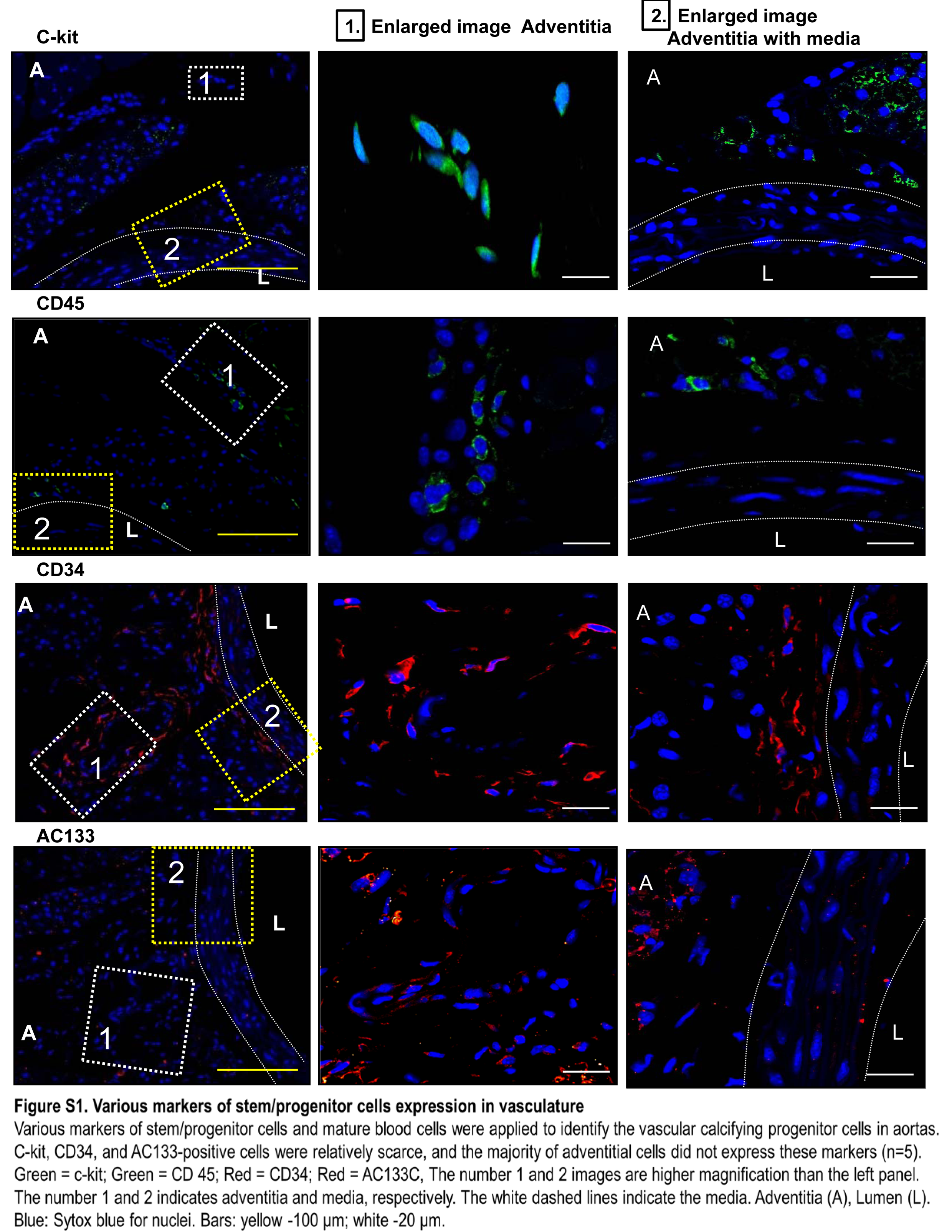

Supplement: Figure S1 — Various markers of stem/progenitor cell expression in vasculature. Various markers of stem/progenitor cells and mature blood cells were applied to identify the vascular calcifying progenitor cells in aortas. C-kit, CD34, and AC133-positive cells were relatively scarce, and the majority of adventitial cells did not express these markers (n = 5). Green, c-kit; green, CD 45; red, CD34; red, AC133C. The number 1 and 2 images are higher magnification than the left panel. The number 1 and 2 indicate adventitia and media, respectively. The white dashed lines indicate the media. A, Adventitia; L, Lumen; blue, Sytox blue for nuclei. Bars: yellow, −100 µm; white, −20 µm. (TIF) [file pbio.1001534.s001.tif]

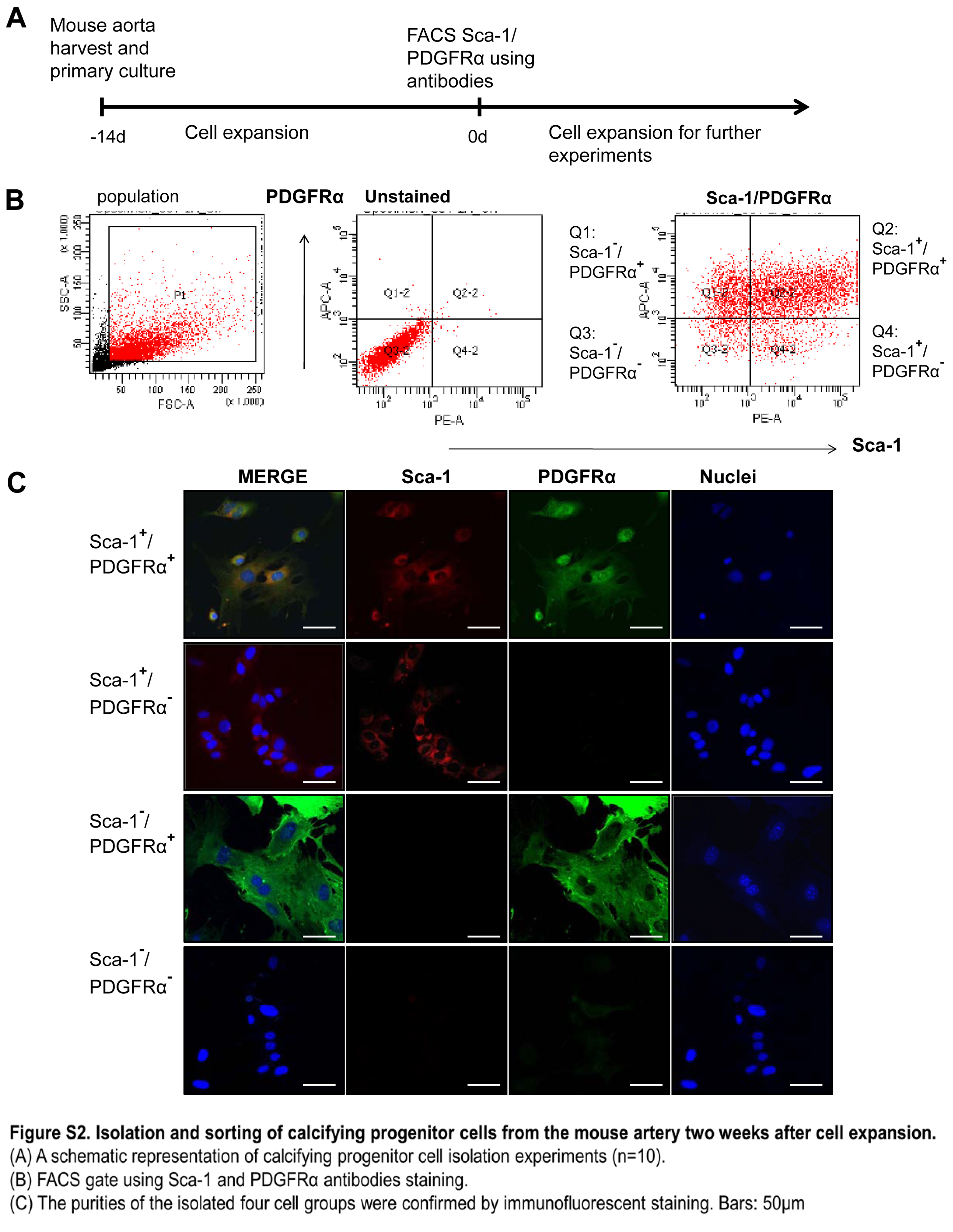

Supplement: Figure S2 — Isolation and sorting of calcifying progenitor cells from the mouse artery 2 wk after cell expansion. (A) A schematic representation of calcifying progenitor cell isolation experiments (n = 10). (B) FACS gate using Sca-1 and PDGFRα antibodies staining. (C) The purities of the isolated four cell groups were confirmed by immunofluorescent staining. Bars: 50 µm. (TIF) [file pbio.1001534.s002.tif]

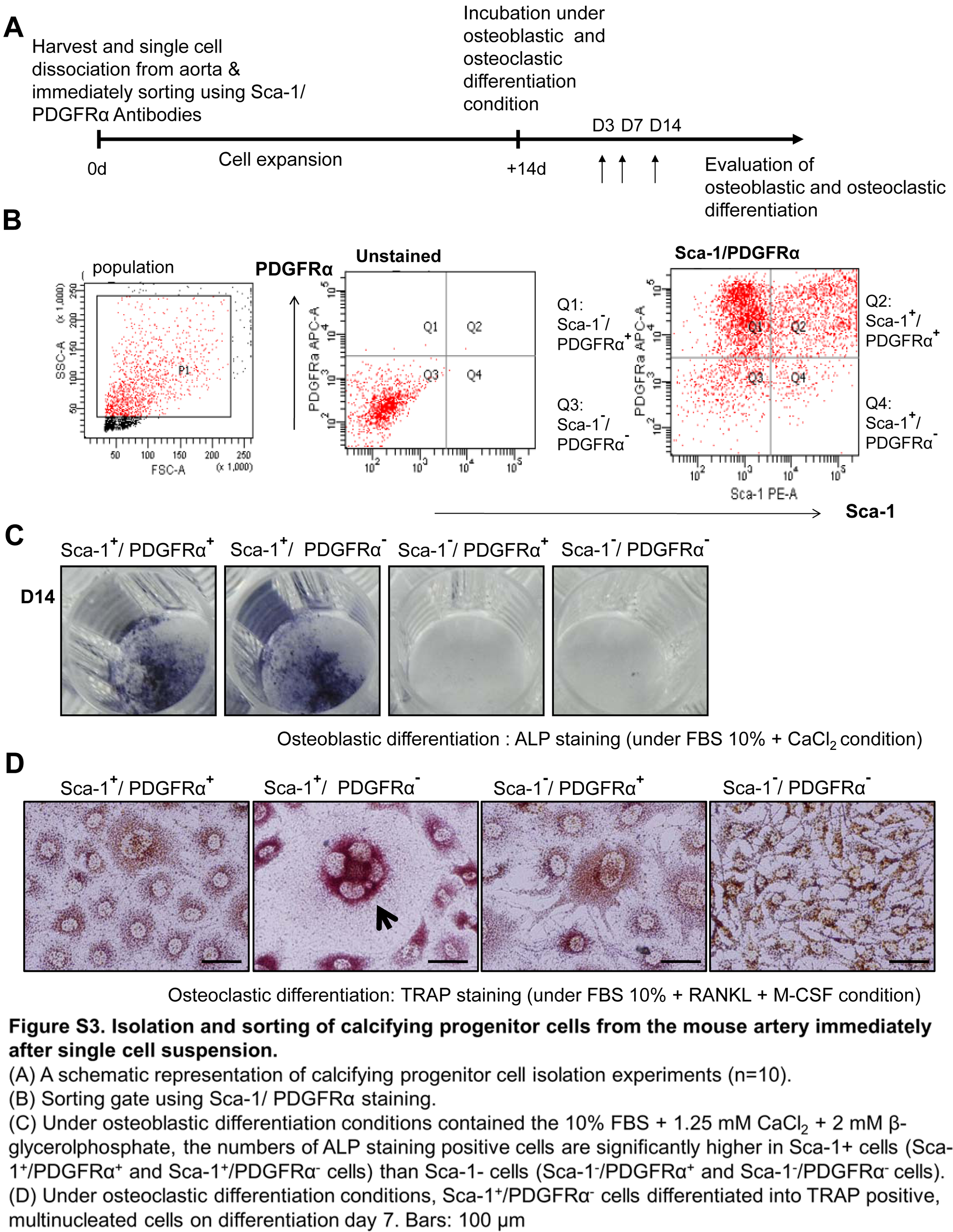

Supplement: Figure S3 — Isolation and sorting of calcifying progenitor cells from the mouse artery immediately after single cell suspension. (A) A schematic representation of calcifying progenitor cell isolation experiments (n = 10). (B) Sorting gate using Sca-1/PDGFRα staining. (C) Under osteoblastic differentiation conditions containing the 10% FBS+1.25 mM CaCl2+2 mM β-glycerolphosphate, the numbers of ALP staining positive cells are significantly higher in Sca-1+ cells (Sca-1+/PDGFRα+ and Sca-1+/PDGFRα− cells) than Sca-1− cells (Sca-1−/PDGFRα+ and Sca-1−/PDGFRα− cells). (D) Under osteoclastic differentiation conditions, Sca-1+/PDGFRα− cells differentiated into TRAP positive, multinucleated cells on differentiation day 7. Bars: 100 µm. (TIF) [file pbio.1001534.s003.tif]

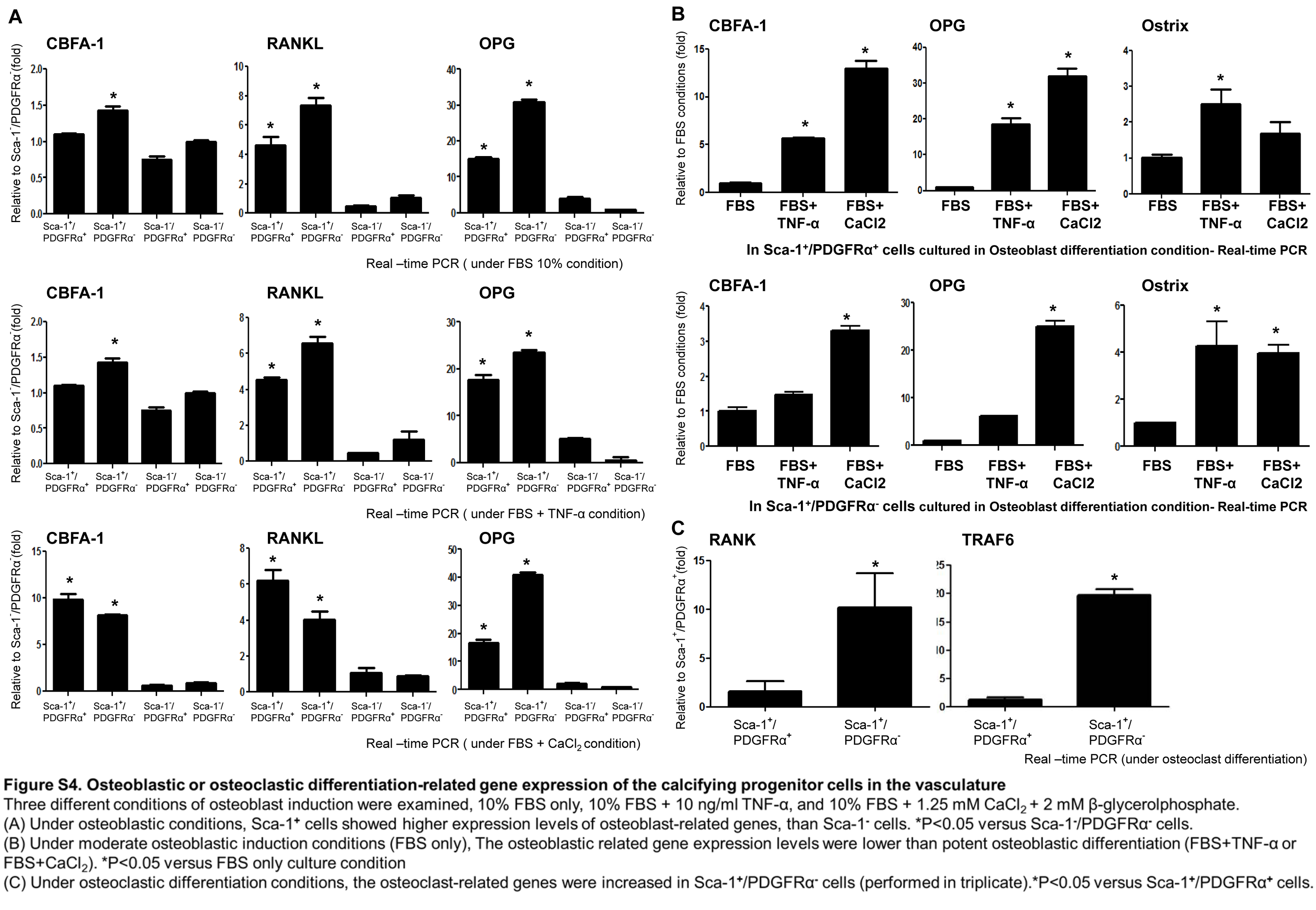

Supplement: Figure S4 — Osteoblastic or osteoclastic differentiation-related gene expression of the calcifying progenitor cells in the vasculature. Three different conditions of osteoblast induction were examined, 10% FBS only, 10% FBS+10 ng/ml TNF-α, and 10% FBS+1.25 mM CaCl2+2 mM β-glycerolphosphate. (A) Under osteoblastic conditions, Sca-1+ cells showed higher expression levels of osteoblast-related genes, than Sca-1− cells on differentiation day 7. *P<0.05 versus Sca-1−/PDGFRα− cells. (B) Under moderate osteoblastic induction conditions (FBS only), the osteoblastic related gene expression levels were lower than potent osteoblastic differentiation (FBS+TNF-α or FBS+CaCl2) on differentiation day 7. *P<0.05 versus FBS only culture condition. (C) Under osteoclastic differentiation conditions, the osteoclast-related genes were increased in Sca-1+/PDGFRα− cells (performed in triplicate) on differentiation day 14. *P<0.05 versus Sca-1+/PDGFRα+ cells. (TIF) [file pbio.1001534.s004.tif]

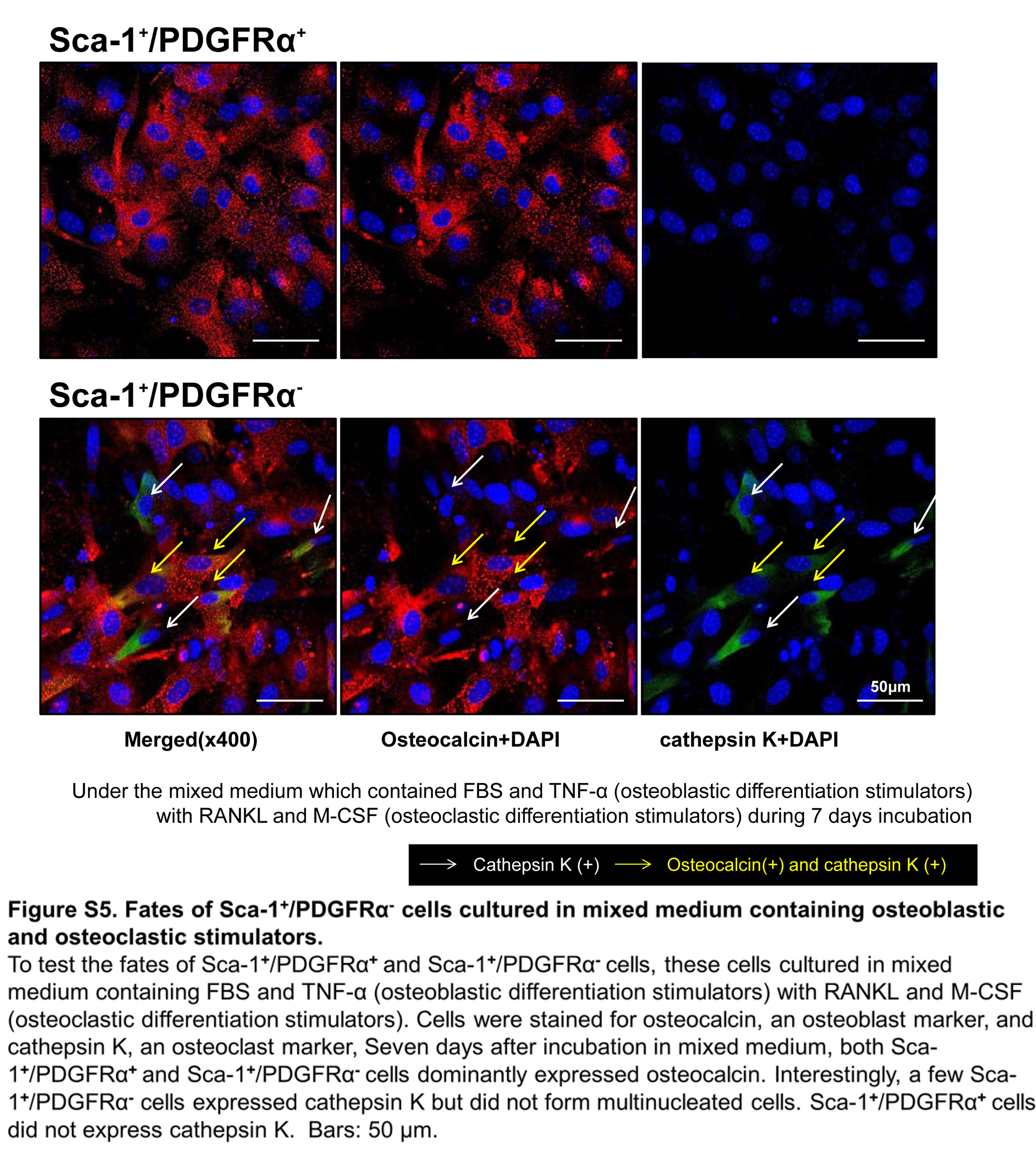

Supplement: Figure S5 — Fates of Sca-1+/PDGFRα− cells cultured in mixed medium containing osteoblastic and osteoclastic stimulators. To test the fates of Sca-1+/PDGFRα+ and Sca-1+/PDGFRα− cells, these cells cultured in mixed medium containing FBS and TNF-α (osteoblastic differentiation stimulators) with RANKL and M-CSF (osteoclastic differentiation stimulators). Cells were stained for osteocalcin, an osteoblast marker, and cathepsin K, an osteoclast marker. Seven days after incubation in mixed medium, both Sca-1+/PDGFRα+ and Sca-1+/PDGFRα− cells dominantly expressed osteocalcin. Interestingly, a few Sca-1+/PDGFRα− cells expressed cathepsin K but did not form multinucleated cells. Sca-1+/PDGFRα+ cells did not express cathepsin K. Bars: 50 µm. (TIF) [file pbio.1001534.s005.tif]

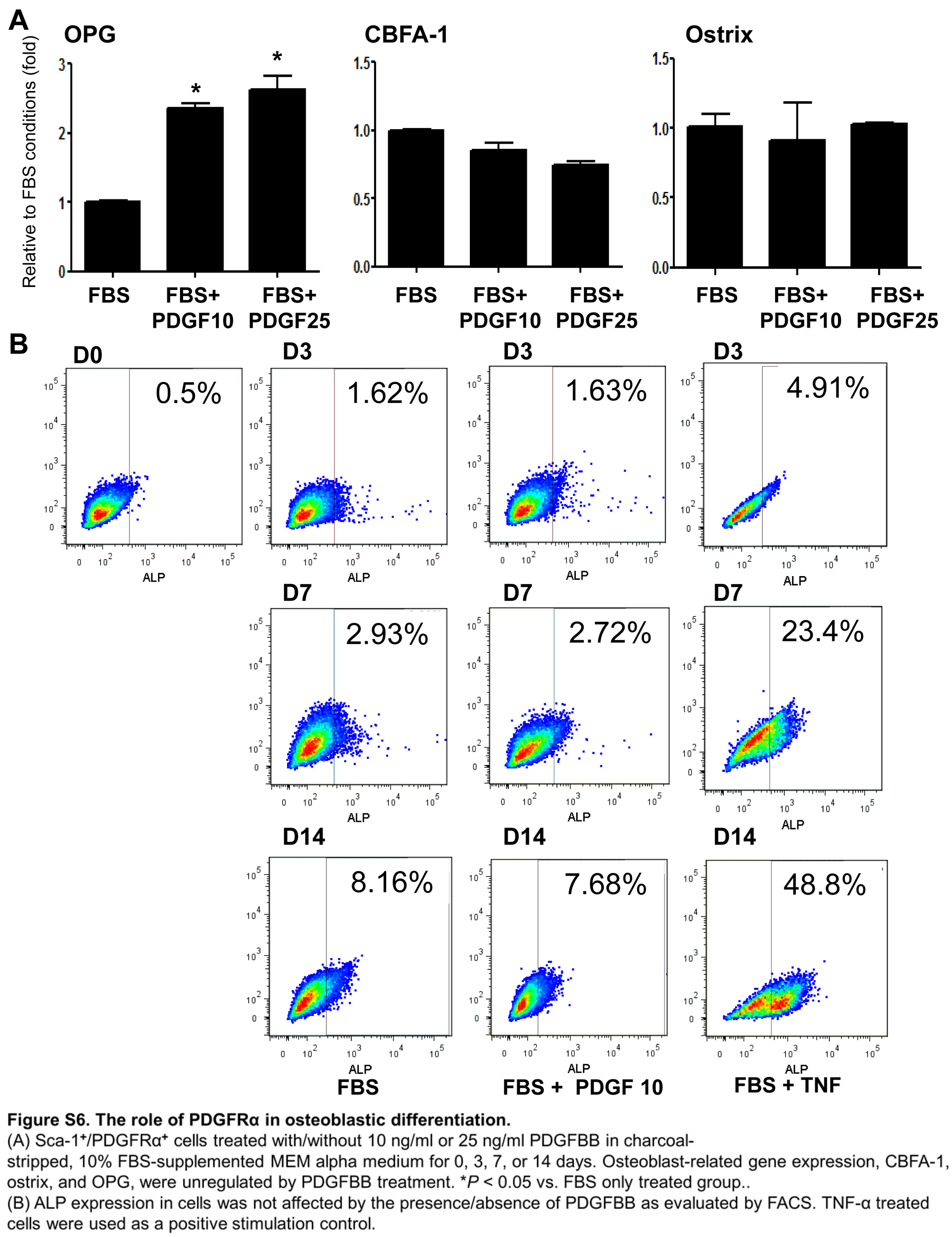

Supplement: Figure S6 — The role of PDGFRα in osteoblastic differentiation. Sca-1+/PDGFRα+ cells treated with/without 10 ng/ml or 25 ng/ml PDGFBB in charcoal-stripped, 10% FBS-supplemented MEM alpha medium for 0, 3, 7, or 14 d. (A) After 7 d, Osteoblast-related gene expression, CBFA-1, ostrix, and OPG were unregulated by PDGFBB treatment. *P<0.05 versus FBS only treated group. (B) ALP expression in cells was not affected by the presence/absence of PDGFBB as evaluated by FACS. TNF-α-treated cells were used as a positive stimulation control. (TIF) [file pbio.1001534.s006.tif]

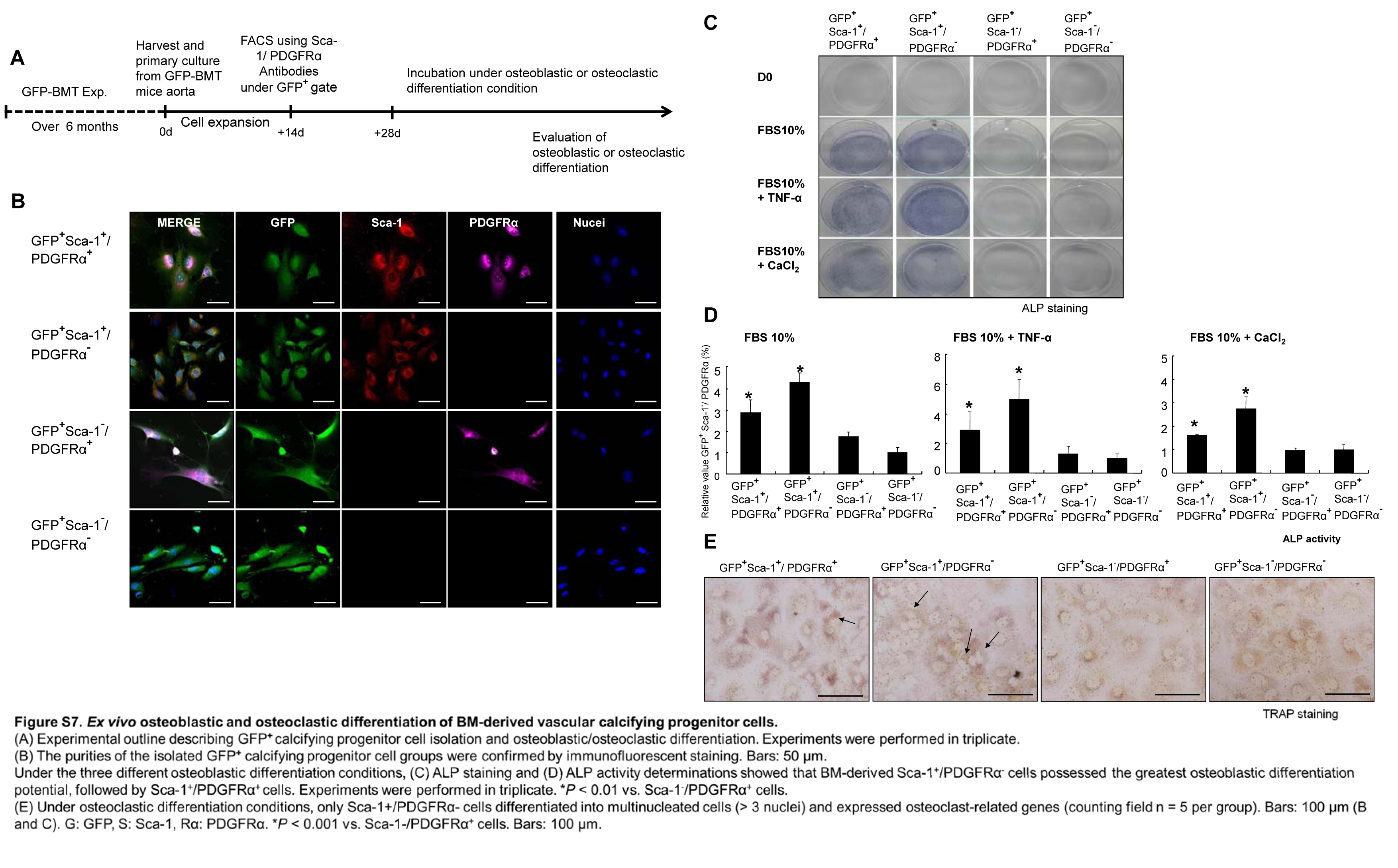

Supplement: Figure S7 — Ex vivo osteoblastic and osteoclastic differentiation of BM-derived vascular calcifying progenitor cells. (A) Experimental outline describing GFP+ calcifying progenitor cell isolation and osteoblastic/osteoclastic differentiation. Experiments were performed in triplicate. (B) The purities of the isolated GFP+ calcifying progenitor cell groups were confirmed by immunofluorescent staining. Bars: 50 µm. Under the three different osteoblastic differentiation conditions, (C) ALP staining and (D) ALP activity determinations showed that BM-derived Sca-1+/PDGFRα− cells possessed the greatest osteoblastic differentiation potential, followed by Sca-1+/PDGFRα+ cells. Experiments were performed in triplicate. *P<0.01 versus Sca-1−/PDGFRα+ cells. (E) Under osteoclastic differentiation conditions, only Sca-1+/PDGFRα− cells differentiated into multinucleated cells (>3 nuclei) and expressed osteoclast-related genes (counting field n = 5 per group). Bars: 100 µm (B and C). G, GFP; S, Sca-1; Rα, PDGFRα. *P<0.001 versus Sca-1−/PDGFRα+ cells. Bars: 100 µm. (TIF) [file pbio.1001534.s007.tif]

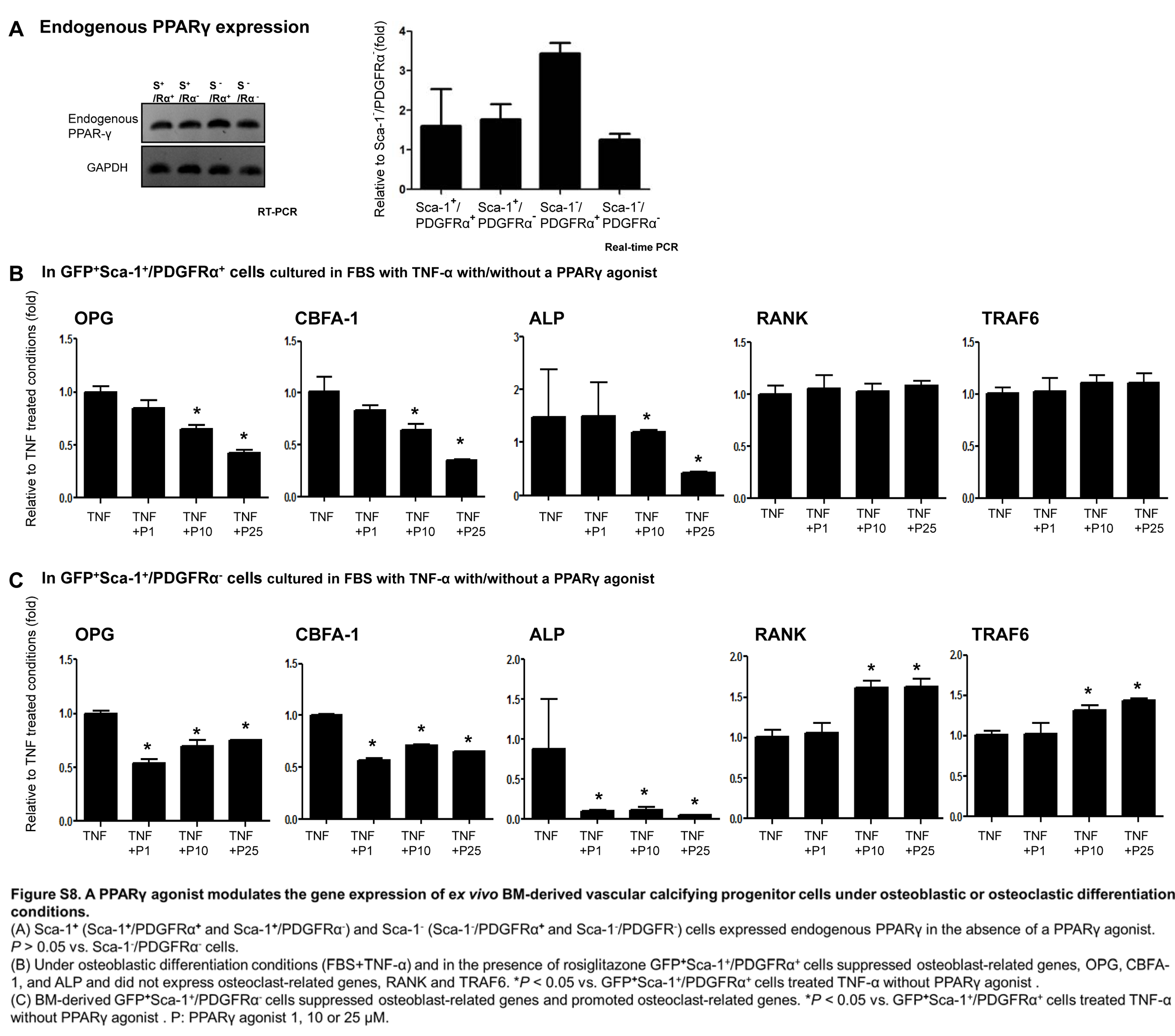

Supplement: Figure S8 — A PPARγ agonist modulates the gene expression of ex vivo BM-derived vascular calcifying progenitor cells under osteoblastic or osteoclastic differentiation conditions. (A) Sca-1+ (Sca-1+/PDGFRα+ and Sca-1+/PDGFRα−) and Sca-1− (Sca-1−/PDGFRα+ and Sca-1−/PDGFR−) cells expressed endogenous PPARγ in the absence of a PPARγ agonist. P>0.05 versus Sca-1−/PDGFRα− cells. (B) Under osteoblastic differentiation conditions (FBS+TNF-α) and in the presence of rosiglitazone for 5 d GFP+Sca-1+/PDGFRα+ cells suppressed osteoblast-related genes, OPG, CBFA-1, and ALP and did not express osteoclast-related genes, RANK and TRAF6. *P<0.05 versus GFP+Sca-1+/PDGFRα+ cells treated TNF-α without PPARγ agonist. (C) BM-derived GFP+Sca-1+/PDGFRα− cells suppressed osteoblast-related genes and promoted osteoclast-related genes. *P<0.05 versus GFP+Sca-1+/PDGFRα+ cells treated TNF-α without PPARγ agonist. P, PPARγ agonist 1, 10, or 25 µM. (TIF) [file pbio.1001534.s008.tif]

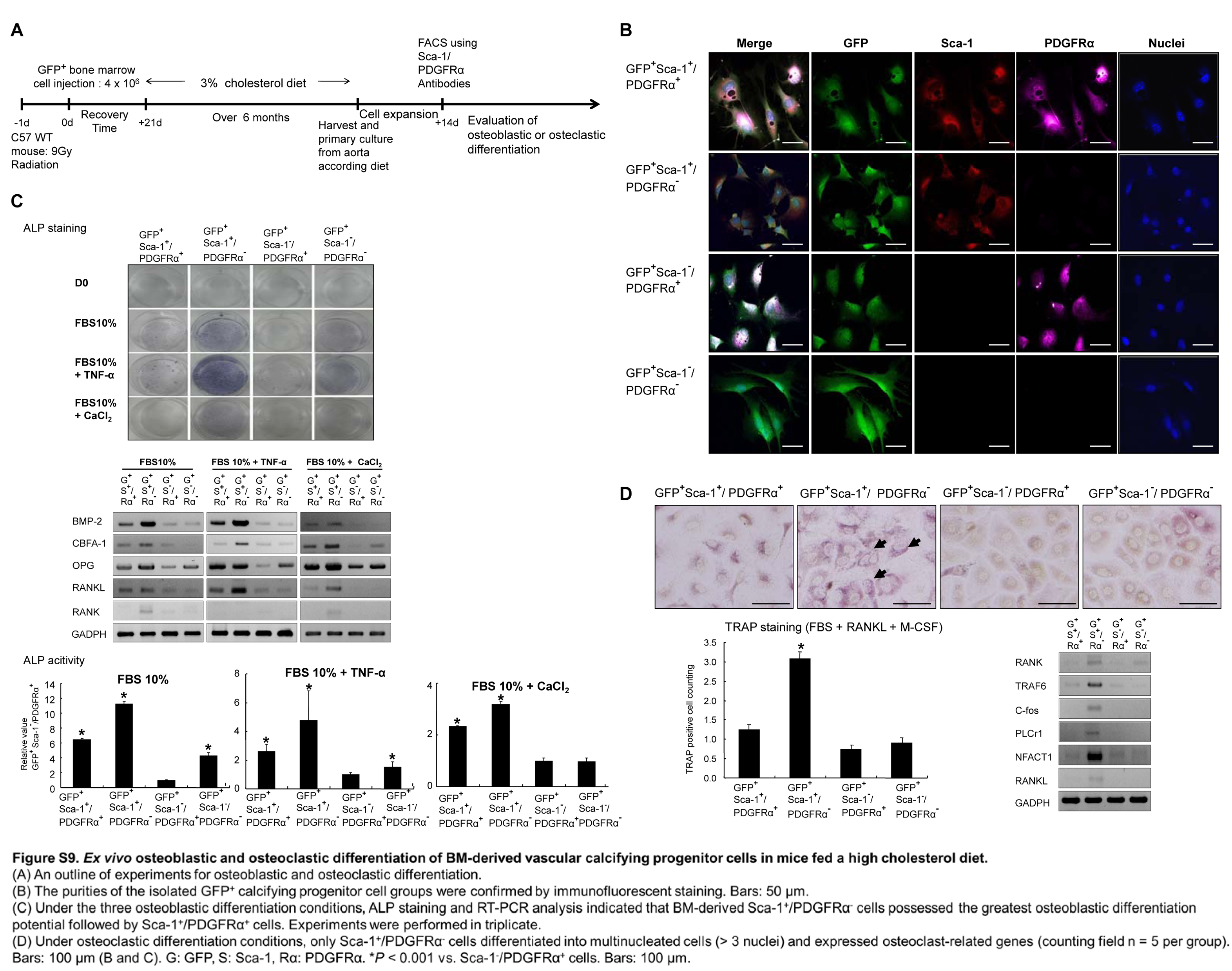

Supplement: Figure S9 — Ex vivo osteoblastic and osteoclastic differentiation of BM-derived vascular calcifying progenitor cells in mice fed a high cholesterol diet. (A) An outline of experiments for osteoblastic and osteoclastic differentiation. (B) The purities of the isolated GFP+ calcifying progenitor cell groups were confirmed by immunofluorescent staining. Bars: 50 µm. (C) Under the three osteoblastic differentiation conditions, ALP staining and RT-PCR analysis indicated that BM-derived Sca-1+/PDGFRα− cells possessed the greatest osteoblastic differentiation potential followed by Sca-1+/PDGFRα+ cells. Experiments were performed in triplicate. (D) Under osteoclastic differentiation conditions, only Sca-1+/PDGFRα− cells differentiated into multinucleated cells (>3 nuclei) and expressed osteoclast-related genes (counting field n = 5 per group). Bars: 100 µm (B and C). G, GFP; S, Sca-1; Rα, PDGFRα. *P<0.001 versus Sca-1−/PDGFRα+ cells. Bars: 100 µm. (TIF) [file pbio.1001534.s009.tif]
