# Supplementary material for: Vascular Calcifying Progenitor Cells Possess Bidirectional Differentiation Potentials
Source: PLoS Biol. 2013 Apr 9;11(4):e1001534. doi: 10.1371/journal.pbio.1001534 (PMC3621676; doi:10.1371/journal.pbio.1001534)
Supplement: Table S1 — Primer sequences of RT-PCR, TMs, and the sizes of PCR products. (DOCX) [file pbio.1001534.s010.docx]

**Table S1.** Primer sequences, TMs, and the sizes of conventional PCR products

| **primer** | **Sequence** | **TM** | **Cycle Number** | **Product size** |
| --- | --- | --- | --- | --- |
| **BMP2** | Forward 5′-CTGTCCCCAGTGACGAGTTT-3′ | 58℃ | 30 | 600bp |
|  | Reverse 5′- AGTTCAGGTGGTCAGCAAGG-3′ |  |  |  |
| **CBFA-1** | Forward 5′-GCGTCTCCACAGAGGATTTC-3′ | 58℃ | 25 | 386bp |
|  | Reverse 5′-CGTCCTGTGAGAGCCATTTC-3′ |  |  |  |
| **OPG** | Forward 5′-CTGCCTGGGAAGAAGAAGATCAG-3′ | 58℃ | 25 | 600bp |
|  | Reverse 5′-TGTGAGGAGAGGAAGGAAGG-3′ |  |  |  |
| **RANKL** | Forward 5′-CATTTGCACACCTCACCATC-3′ | 56℃ | 30 | 399bp |
|  | Reverse 5′-AAGGGTTGGACACCTGAATG-3′ |  |  |  |
| **RANK** | Forward 5′-AAACCTTGGACCAACTGCAC-3′ | 56℃ | 30 | 584bp |
|  | Reverse 5′-TTGCTTCCCTGCTGGATTAG-3′ |  |  |  |
| **NFATc1** | Forward 5′-GTCTCTCCTCCAGAAGCTGT-3′ | 58℃ | 25 | 488bp |
|  | Reverse 5′-AGAGTGCTATCGGTGGTCAG-3′ |  |  |  |
| **PLCγ1** | Forward 5′-ATCAAGAGAGCGCTGGAGTT-3′ | 56℃ | 25 | 437bp |
|  | Reverse 5′-AGGCTGATGCTGTTTCTTCG-3′ |  |  |  |
| **TRAF6** | Forward 5′-CACTCATGCAGGCAAAAC-3′ | 56℃ | 30 | 483bp |
|  | Reverse 5′-GAAACAGACTCCTTGGTGGA-3′ |  |  |  |
| **c-Fos** | Forward 5′-GGCTTCAACGCAGACTACGAGG-3′ | 58℃ | 30 | 340bp |
|  | Reverse 5′-CTCCTGTCATGGTCTTCACAACG-3′ |  |  |  |
| **GAPDH** | Forward 5′-CGTGGAAGGACTCATGAC-3′ | 58℃ | 25 | 500bp |
|  | Reverse 5′-CAAATTCGTTGTCATACCAG-3 |  |  |  |
