# Supplementary material for: Vascular Calcifying Progenitor Cells Possess Bidirectional Differentiation Potentials
Source: PLoS Biol. 2013 Apr 9;11(4):e1001534. doi: 10.1371/journal.pbio.1001534 (PMC3621676; doi:10.1371/journal.pbio.1001534)
Supplement: Table S2 — Primer sequences for osteoblast and osteoclast marker of real-time PCR. (DOCX) [file pbio.1001534.s011.docx]

**Table S2.** Primer sequences for osteoblast and osteoclast marker of real-time PCR.

| **primer** | **Sequence** |
| --- | --- |
| **CBFA-1** | Forward 5′- TGTTCTCTGATCGCCTCAGTG -3′ |
|  | Reverse 5′- CCTGGGATCTGTAATCTGACTCT -3′ |
| **RANKL** | Forward 5′- CCAGCTATGATGGAAGGCTCA -3′ |
|  | Reverse 5′- CGTACAGGTAATAGAAGCCA -3′ |
| **OPG** | Forward 5′- GGGCGTTACCTGGAGATCG -3′ |
|  | Reverse 5′- GAGAAGAACCCATCTGGACATTT -3′ |
| **Ostrix** | Forward 5′- ATGGCGTCCTCTCTGCTTG -3′ |
|  | Reverse 5′- TGAAAGGTCAGCGTATGGCTT -3′ |
| **ALP** | Forward 5′- ATGGGCGTCTCCACAGTAAC -3′ |
|  | Reverse 5′- CTTCACGCCACACAAGTAGG -3′ |
| **Osteocalcin** | Forward 5′- CCTGCTTGTGACGAGCTAT -3′ |
|  | Reverse 5′- ACTTGCAGGGCAGAGAGAGA -3′ |
| **RANK** | Forward 5′- CTGGGAACGTGACTGGAAAC -3′ |
|  | Reverse 5′- CCGCAAAGGAGTCTCTGTGT -3′ |
| **TRAF6** | Forward 5′- ACAGGCAGACCTGGGTACAA -3′ |
|  | Reverse 5′- GTCCATGACCTCTTCGTGGT -3′ |
| **GAPDH** | Forward 5′- TCTCCATGGTGGTGAAGACA-3′ |
|  | Reverse 5′- ACTCCACTCACGGCAAATTC-3 |
