# Supplementary material for: Vascular Calcifying Progenitor Cells Possess Bidirectional Differentiation Potentials
Source: PLoS Biol. 2013 Apr 9;11(4):e1001534. doi: 10.1371/journal.pbio.1001534 (PMC3621676; doi:10.1371/journal.pbio.1001534)
Supplement: Table S3 — Primer sequences for adipocyte and chondrocyte marker of real-time PCR. (DOCX) [file pbio.1001534.s012.docx]

**Table S3.** Primer sequences for adipocyte and chondrocyte marker of real-time PCR.

| **primer** | **Sequence** |
| --- | --- |
| **PPARγ** | Forward 5′- TCAGCTCTGTGGACCTCTCC -3′ |
|  | Reverse 5′- ACCCTTGCATCCTTCACAAG -3′ |
| **C/EBPα** | Forward 5′- CAAGAACAGCAACGAGTACCG -3′ |
|  | Reverse 5′- GTCACTGGTCAACTCCAGCAC -3′ |
| **C/EBPβ** | Forward 5′- CCAAGAAGACGGTGGACAA -3′ |
|  | Reverse 5′- CAAGTTCCGCAGGGTGCT -3′ |
| **Collagen 1a1** | Forward 5′- GCAACAGTCGCTTCACCTAC -3′ |
|  | Reverse 5′- GTGGGAGGGAACCAGATTG -3′ |
| **Collagen 2a1** | Forward 5′- TTGAGACAGCACGACGTGGAG -3′ |
|  | Reverse 5′- AGCCAGGTTGCCATCGCCATA -3′ |
| **Aggrecan** | Forward 5′- GCGAGTCCAACTCTTCAAGC -3′ |
|  | Reverse 5′- GAAGTAGCAGGGGATGGTGA -3′ |
| **GAPDH** | Forward 5′- TCTCCATGGTGGTGAAGACA-3′ |
|  | Reverse 5′- ACTCCACTCACGGCAAATTC-3 |
